# Characterization of the salivary microbiome in people with obesity

**DOI:** 10.7717/peerj.4458

**Published:** 2018-03-16

**Authors:** Yujia Wu, Xiaopei Chi, Qian Zhang, Feng Chen, Xuliang Deng

**Affiliations:** 1 Department of Geriatric Dentistry, Peking University School and Hospital of Stomatology, Beijing, China; 2 Department of Prosthodontics, Peking University School and Hospital of Stomatology, Beijing, China; 3 Central Laboratory, Peking University School and Hospital of Stomatology, Beijing, China

**Keywords:** Obesity, Body-Mass Index, High-throughput nucleotide sequencing, Oral microbiome, Microbiome

## Abstract

**Background:**

The interactions between the gut microbiome and obesity have been extensively studied. Although the oral cavity is the gateway to the gut, and is extensively colonized with microbes, little is known about the oral microbiome in people with obesity. In the present study, we investigated the salivary microbiome in obese and normal weight healthy participants using metagenomic analysis. The subjects were categorized into two groups, obesity and normal weight, based on their BMIs.

**Methods:**

We characterized the salivary microbiome of 33 adults with obesity and 29 normal weight controls using high-throughput sequencing of the V3–V4 region of the 16S rRNA gene (Illumina MiSeq). None of the selected participants had systemic, oral mucosal, or periodontal diseases.

**Results:**

The salivary microbiome of the obesity group was distinct from that of the normal weight group. The salivary microbiome of periodontally healthy people with obesity had both significantly lower bacterial diversity and richness compared with the controls. The genus *Prevotella, Granulicatella*, *Peptostreptococcus, Solobacterium, Catonella*, and *Mogibacterium* were significantly more abundant in the obesity group; meanwhile the genus *Haemophilus, Corynebacterium, Capnocytophaga*, and *Staphylococcus* were less abundant in the obesity group. We also performed a functional analysis of the inferred metagenomes, and showed that the salivary community associated with obesity had a stronger signature of immune disease and a decreased functional signature related to environmental adaptation and Xenobiotics biodegradation compared with the normal weight controls.

**Discussion:**

Our study demonstrates that the microbial diversity and structure of the salivary microbiome in people with obesity are significantly different from those of normal weight controls. These results suggested that changes in the structure and function of salivary microbiome in people with obesity might reflect their susceptibility to oral diseases.

## Introduction

Obesity and its co-morbidities threaten the health of humans worldwide. The prevalence of obesity amongst Chinese adults has increased from 3.8% to 11.3% over the past two decades (Mi et al., 2015). Thus, based on the large population, China now contains the second largest population of people with obesity after the United States of America (Ng et al.,  2014).

There is a growing interest in the impact of the gut microbiome on host physiology and health. Quantitative and qualitative alterations in gut microbiome composition and diversity can lead to pathological dysbiosis and are important predictors of many diseases (Hollister, Gao & Versalovic, 2014). It has been shown that an altered gut microbiome is associated with obesity and its co-morbidities, such as type 2 diabetes (Backhed et al., 2004; Turnbaugh et al., 2006; Devaraj, Hemarajata & Versalovic, 2013; Ridaura et al., 2013; Nieuwdorp et al., 2014). Some researchers have proposed that certain gut microbiota compositions, along with an environmental predispositions, can lead to obesity via an impairment in energy homeostasis resulting in metabolic disease(s) (Moreno-Indias et al., 2014). Studies characterized the composition and diversity of the gut microbiome in lean and obese phenotypes, and showed that mice with obesity had a lower gut microbial diversity as well as an altered distributions of microbiota compared with the lean mice (Turnbaugh et al., 2006; Turnbaugh et al., 2009). Human studies also evaluated the gut microbiota in obese individuals, and demonstrated a higher ratio of Firmicutes to Bacteroidetes (F/B) in the gut of obese individuals (Ley et al., 2006). However, some others produced the opposite conclusion or failed to find significant differences in the F/B ratio between the obesity and the controls (Duncan et al., 2008; Furet et al., 2010; Jumpertz et al., 2011). Researchers then suggested that the gut microbiota can be classified into three robust enterotypes (Bacteroides, Prevotella, or Ruminococcus)*,* which correlate with body mass index (Arumugam et al., 2011). Later studies reduced the enterotypes to two clusters: prevotella or Bacteroides dominated. An altered gut microbial community may contribute to the development of obesity in the host through mechanisms including increased energy harvest from the diet, chronic low-grade endotoxinemia, regulation of fatty acid metabolism, and modulation of gut-derived peptide secretion (Musso, Gambino & Cassader, 2010).

The oral cavity is one of the most clinically relevant microbial habitats. Several studies have shown that oral bacteria contribute to oral diseases (e.g., dental caries, periodontitis, halitosis), and are also significant risk factor for diabetes mellitus, preterm birth, cardiovascular diseases, bacteremia, and tumors (He et al., 2015). Furthermore, the salivary microbiome is altered in patients suffering from systemic diseases, such as liver cirrhosis, rheumatoid arthritis, and pancreatic cancer; these changes are also reflected in the gut microbiome (Farrell et al., 2012; Qin et al., 2014; Zhang et al., 2015; Torres et al., 2015). However, little attention has been paid to the overall characterization of the salivary microbiome in people with obesity. A link between the oral microbiome and obesity was first made in 2009 (Goodson et al., 2009). Several studies have reported differences in the oral microbiomes of people with obesity and normal weight people, but the data are inconsistent. In the oral subgingival biofilms of adolescents with obesity, researchers discovered a higher cellular abundance of bacteria with a high phylogenetic diversity (Zeigler et al., 2012). Piombino et al. (2014) found microbiological and biochemical differences between the saliva of people with obesity and those of normal weight. Most recently, a study of the salivary microbiome of Japanese adults found a higher phylogenetic diversity in individuals with obesity; however, the authors noted that the effects of periodontal disease might have confounded the results (Takeshita et al., 2016). The interactions between the salivary microbiome and obesity are still unclear, and more studies are needed to evaluate the changes in saliva microbiota composition and the link to metabolic status.

To obtain a complete picture of the oral microbiome of people with obesity, we used high-throughput sequencing of the 16S rRNA gene to compare the salivary microbiome in periodontally healthy adults with obesity with normal weight controls.

## Materials and Methods

### Participant recruitment and sample collection

The study was approved by the Ethics Committee of Peking University School and the Hospital of Stomatology (PKUSSIRB- 201627023), and written informed consent was obtained from all participants. We recruited 40 people with obesity and 40 age- and sex-matched people of normal weight as controls. Each individual completed a basic questionnaire and was given a comprehensive oral examination. Decayed, missing, and filled teeth (DFMT); decayed, missed, filled surfaces (DMFS); plaque index (PLI); sulcus bleeding index (SBI), periodontal pocket depth (PPD); number of teeth, and number of restorations were examined. A single experienced dentist performed all of the examinations and diagnoses. The inclusion criteria were as follows: aged between 20 and 40 years, the body mass index (BMI) was calculated and the participants were divided into a normal weight group (BMI = 18.5–20) and an obesity group (BMI ≥30), based on the WHO guidelines, mean periodontal pocket depth ≤3 mm, and less than 10% of teeth which sulcus bleeding index ≥3. The exclusion criteria were: diagnosed presence of a disease, use of medications, a history of smoking, pregnancy/lactation, menopause, use of antibiotics, hormonal contraceptives within the three months prior to sample collection, fewer than 20 teeth in the oral cavity, or any periodontal attachment loss. Subjects were divided into group based on body mass index (BMI), with subjects having a BMI between 18.5 and 20 being placed in the normal weight group and subjects having a BMI greater than 30 being placed in the obesity group; this criterion is based on WHO guidelines. We screened 80 persons; 33 people with obesity and 29 normal weight people in good oral health were enrolled in the study. Eighteen subjects were excluded by their having periodontitis or less than 20 teeth.

Saliva samples were collected according to a standard technique: 5 mL of spontaneous, whole, unstimulated saliva was collected into a 50 mL sterile DNA-free conical tube from each participant between 8–11 a.m. (maximum collection time fixed at 30 min). Participants were asked not to drink or eat for at least 8 h before sampling. The samples were divided into five aliquots and stored at −80 °C until further analysis.

### DNA extraction and 16S rRNA gene pyrosequencing

Genomic DNA was extracted using the QIAamp DNA Blood Mini Kit according to the manufacturer’s instructions (Qiagen, Hilden, Germany). DNA purity was evaluated via A260/A280 ratio using a NanoDrop 7000 Spectrophotometer (Thermo Fisher Scientific, Waltham, MA, USA), and DNA integrity was checked by 1% agarose gel electrophoresis. PCR amplification of the V3–V4 regions of bacterial 16S rRNA was performed using the universal primers (338F 5′-ACTCCTACGGGAGGCAGCA-3′ and 806R 5′-GGACTACHVGGGTWTCTAAT-3′). All PCR products were sequenced on an Illumina Miseq PE300 Sequencing platform according to the standard protocols (Illumina, Inc., San Diego, CA, USA). The PCR program was: initial denaturation at 95 °C for 5 min; 25 cycles of denaturation at 95 °C for 30 s, annealing at 56 °C for 30 s, and extension at 72 °C for 40 s, and; final extension of 72 °C for 10 min. The amplicon mixture were pooled according to the manufacturer’s instructions (Illumina, Inc., San Diego, CA, USA). The sequence data had been deposited the NCBI GenBank database (http://www.ncbi.nlm.nih.gov) under the accession number MF800965–MF801301.

### 16S data processing and statistical analysis

A sample size estimation was performed to determine the probability that the samples were representative (Motulsky, 2010). The raw sequencing data were analyzed using the Quantitative Insights Into Microbial Ecology (QIIME v.1.8.1; http://www.qiime.org) package (Caporaso et al., 2010). Paired V3–V4 16S rRNA sequences were trimmed using trimmomatic (Version 0.33) software and merged into a single sequence using FLASH (Version 1.2.10). Merged sequences were filtered to remove the low-quality sequences and binned according to their specific barcodes. Sequences were assembled according to the following criteria: (i) raw reads shorter than 110 nucleotides were discarded; (ii) reads were truncated at any site receiving an average quality score <20 over a 50 bp sliding window, and the truncated reads that were shorter than 50 bp were discarded; and, (iii) only sequences that overlapped for more than 10 bp were assembled. Reads that could not be assembled were discarded. The unique sequence set was classified into operational taxonomic units (OTUs) with a cutoff of 97% identity using the *de novo* OTU selection strategy. We retained only those OTUs that were present with at least 0.01% mean relative abundance as predominant. Taxonomies were assigned by the RDP classifier (Version 2.2) (Cole et al., 2003) against the Human Oral Microbiome Database (Chen et al., 2010) (HOMD RefSeq, Version 13.2) with a confidence threshold of 0.7. Chimeric sequences were removed using Usearch (Version 8.0). Data were rarefied to 35,101 reads per sample.

Alpha diversity was assessed by the Chao 1 and Shannon index. To visualize the beta diversity between groups, the unweighted and weighted UniFrac distances (Lozupone, Hamady & Knight, 2006) were calculated and plotted *via* principal coordinate analysis (PCoA). PERMANOVA was used to test significance. Mann–Whitney *U* tests were used for alpha diversity comparisons. Differences in the relative abundances of taxa at the class, order, family, genus levels were analyzed using the Kurskal–Wallis test and plotted using GraphPad Prism (Version 6.0). Adonis testing was used to confirm significant differences in microbial community composition; all of these were calculated in QIIME. All remaining statistical calculations were performed in IBM SPSS Statistics (Version 20) using Mann–Whitney *U* tests to compare between groups. Fisher’s exact tests were used to compare parameters. All statistical tests were two-sided, and *P* values <0.05 were considered to be significant. Benjamini–Hochberg false discovery rate (FDR) correction was used to correct for multiple hypothesis testing where applicable.

Predictive function analysis was performed using the PICRUSt algorithm (Langille et al., 2013) based on the Kyoto Encyclopedia of Genes and Genomes (KEGG) Orthology (KO) classification (Kanehisa et al., 2008). The 16S rRNA gene data was generated using the closed-reference OTUs picked by QIIME over the same set of sequeces against the Greengenes database (Version 18.3). The ‘DESeq’ function in DESeq2 R package was used to detect functional pathways that were significantly different in abundance between groups. Inferred functional shifts in the salivary microbiome and genus-level taxonomic contributions were obtained using the Functional Shifts’ Taxonomic Contributors (FishTaco) software (Manor & Borenstein, 2017). FishTaco was used to infer a taxonomic and functional abundance profile from the PICRUSt analysis. The metagenome- and taxa-based functional shifts were calculated using a comparative functional analysis between samples from the normal weight and obese groups; then, the functional shifts were decomposed into genus-level contributions. Each functional shift in pairwise comparisons with the controls was grouped into one of four different modes: (1) case-associated taxa increasing a functional shift (i.e., case-associated taxa driving case-enrichment); (2) case-associated taxa attenuating case-enrichment (i.e., taxa over-represented in case but no enzymatic activity in the pathway); (3) control-associated taxa driving case-enrichment (i.e., taxa over-represented in controls with no enzymatic activity in the pathway), and; (4) control-associated taxa attenuating case-enrichment (i.e., taxa over-represented in controls but no enzymatic activity in the pathway). The output result was visualized using FishTacoPlot package (https://github.com/borenstein-lab/fishtaco-plot) in R (Version 3.4.3).

## Results

### Demographic and clinical parameters

There were no significant differences in gender distribution; ages; periodontal conditions; tooth-brushing habits; decayed, missing or filled teeth; decayed, missing, or filled surfaces; number of teeth; number of restorations; plaque index; sulcus bleeding index, or tooth-brushing frequency between the two groups (*P* > 0.05) (Table 1). The mean probing depth was greater in participants with obesity than in controls.

**Table 1:** Demographic and clinical characteristics. All of the examinations and diagnoses were performed by a single experienced dentist.

Variables	Obesity (*n* = 33) Mean (SD)	Controls (*n* = 29) Mean (SD)	*P*-value
Male/female	28/5	23/6	0.741^a^
Age, years	31.0(5.5)	29.5(4.0)	0.209^b^
BMI, kg/m^2^	32.8(3.8)	19.7(1.4)	0.000^b^
DMFT	1.2(1.4)	1.4(2.8)	0.053^b^
DMFS	1.4(1.9)	2.8(3.6)	0.061^b^
Number of teeth	27.8(0.8)	27.6(0.9)	0.362^b^
Number of restorations	0.2(0.4)	0.7(2.2)	0.178^b^
Mean PLI	0.8(0.3)	0.8(0.3)	0.595^b^
Mean SBI	1.8(0.4)	1.8(0.4)	0.748^b^
Mean PD	2.8(0.2)	2.6(0.3)	0.010^b^
Tooth-brushing frequency			
More than once a day	21	25	
Once a day	9	4	0.07^c^
Less than once a day	3	0	0.07^c^

**Notes.**

DMFT decayed missing, filled teeth DMFS decayed, missing, filled surfaces PLI plaque index SBI sulcus bleeding index PPD periodontal pocket depth

a *χ*^2^ test.

b ANOVA test.

c Fisher’s exact test.

### Sequencing data

A total of 7,968,621 raw sequence reads were generated from the 62 saliva samples. There were 5,627,309 sequence reads after data trimming and quality filtering, and the average number of reads per sample was 90,763 (ranging from 35,101 to 257,477; Table S1). The average sequence length was 466 bp. 344 OTUs (abundance >0.01%) were detected as predominant, with an average of 222 OTUs per sample (range: 160 to 265).

### The salivary microbiome in people with obesity has a lower bacterial richness than in normal weight controls

**Figure 1:**
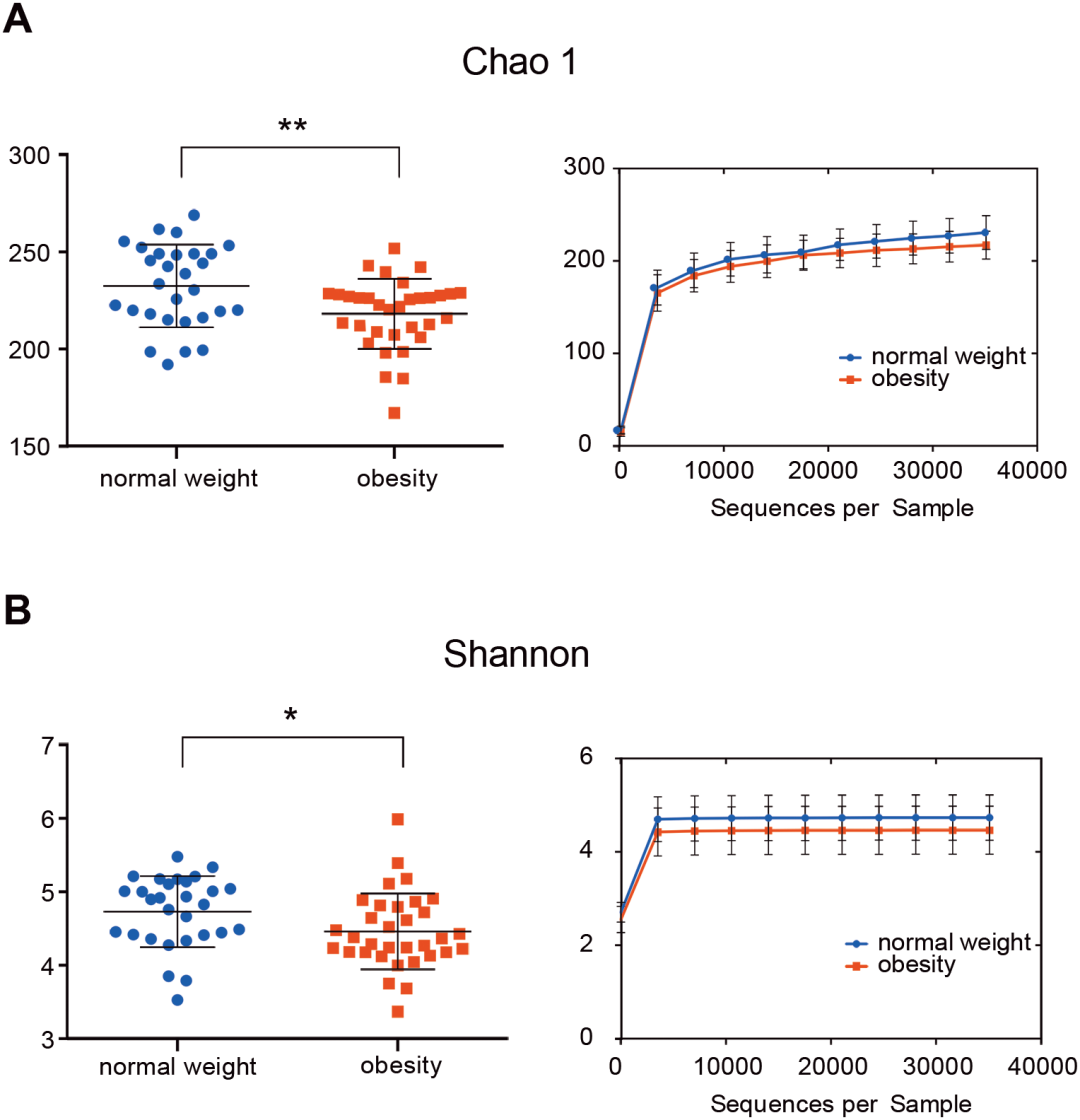
The alpha diversity of people with obesity and controls. The alpha diversity of people with obesity and normal weight controls. A two-tailed Mann–Whitney *U* test was used; ^∗^*P* < 0.05, ^∗∗^*P* < 0.01. Normal weight subjects were significantly different by the Chao 1 (A) and Shannon index (B) measures of diversity.

The indices Chao1, Good’s coverage, observed OTUs, Shannon index, and phylogenetic diversity whole tree were used to examine alpha diversity (Table S2). Bacterial community richness (Chao1) and diversity (Shannon) of the microbiome in the obesity group were lower than in control group (*P* = 0.006 for Chao1 and *P* = 0.037 for Shannon; Fig.  1). The Good’s coverage estimator for each group was >99%, this indicates that the sequencing depth was sufficient to saturate the bacterial diversity. The rank-abundance curve had a steep slope (Fig. S1).

**Figure 2:**
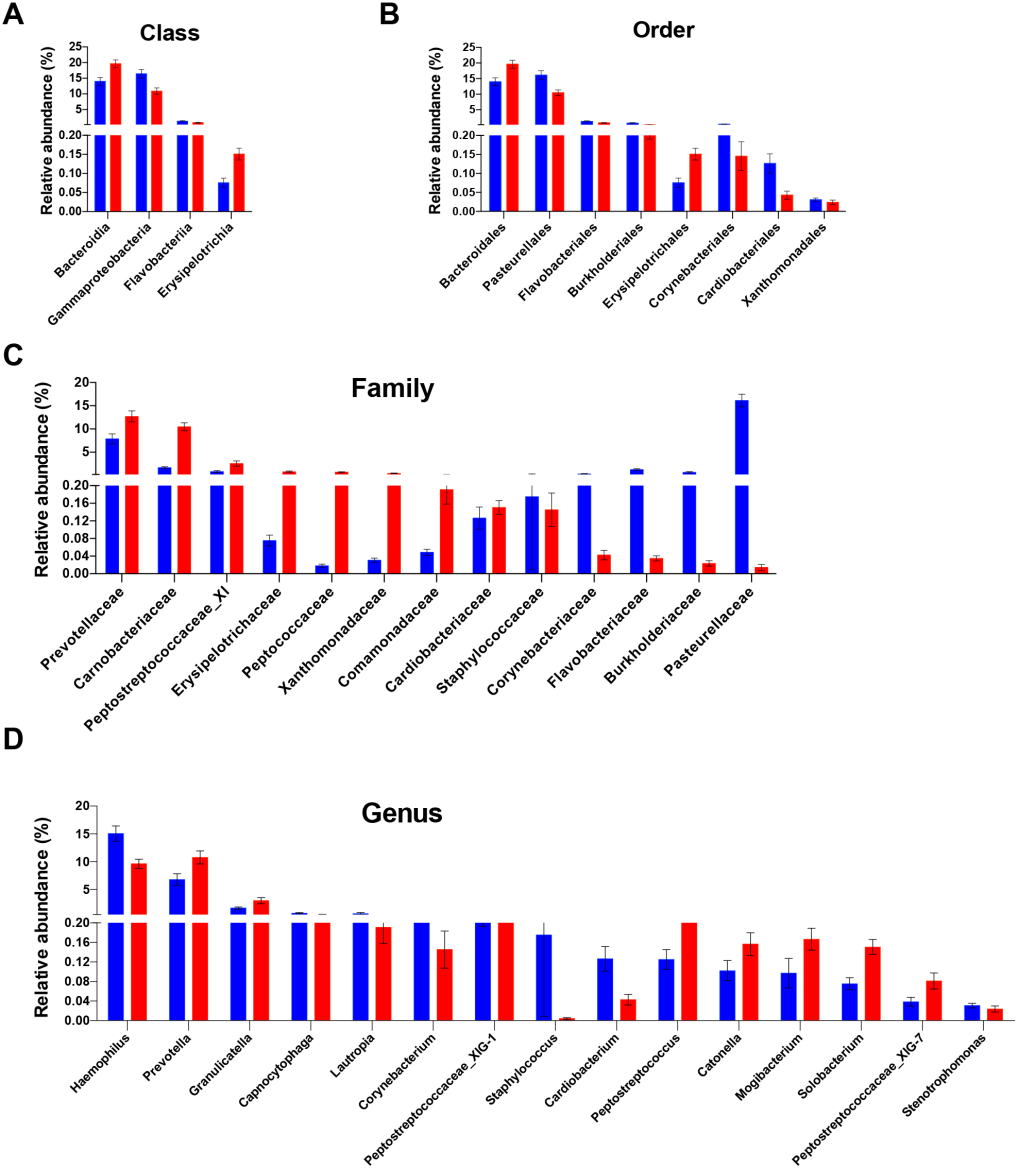
Differentially abundant taxa between people with obesity and normal weight controls. The taxa differed in terms of relative abundance at the class level (A), the order level (B), the family level (C), and the genus level (D). (Kurskal-Wallis test, FDR -adjusted *P* < 0.05). The bars indicate mean ± SEM. Red for obesity group, blue for normal weight group.

### The relative abundance and bacterial community structure of the salivary microbiome differ between people with obesity and normal weight controls

Analysis of the relative abundance of microbial taxonomic groups showed that salivary bacterial composition differed between the two groups. In total, 10 phyla, 20 classes, 27 orders, 39 families, 74 genera, and 181 species were identified in the saliva samples. The five most abundant phyla were Firmicutes (38.8%), Proteobacteria (33.4%), Bacteroidetes (18.0%), Actinobacteria (5.4%), and Fusobacteria (2.9%), which accounted for 98.6% of the total sequences. Other dominant taxa are described in Tables S3–S7. The salivary microbiome was dominated by twelve genera: *streptococcus, Neisseria, Haemophilus, Prevotella, Porphyromonas, Veillonella, Gemella, Rothia, Granulicatella, Fusobacterium, Actinomyces*, and *Alloprevotella*. These genera accounted for 90.2% of all sequences. No other genera had a relative abundance >1%.

The differences in the overall composition of the oral microbiome of the normal weight controls and obese participants were investigated. No statistically significant differences in bacterial relative abundance between the groups were observed at phylum level. The relative abundance levels of four classes, eight orders, and 13 families were significantly different between the two groups. Of these, two classes, two orders, and eight families were over-represented in obese group (FDR adjusted *P* < 0.05, Kruskal–Wallis test; Figs. 2A–2C). At the class level, the proportions of Erysipelotrichia and Bacteroidia were increased in the saliva samples of obese group; meanwhile, the proportions of the Gammaproteobacteria and Flavobacteriia members were decreased (Fig. 2A). At the order level, the proportions of the Bacteroidales, and Erysipelotrichales members were increased in the saliva samples of the obese group; meanwhile, the proportions of the Pasteurellales, Burkholderiales, Flavobacteriales, Corynebacteriales, Cardiobacteriales, and Xanthomonadales were decreased (Fig. 2B). At the family level, Prevotellaceae, Carnobacteriaceae, Peptostreptococcaceae_XI, Erysipelotrichaceae, Peptococcaceae, Xanthomonadaceae, Comamonadaceae, and Cardiobacteriaceae were enriched in the saliva samples of the obese group; meanwhile, the propotions of Pasteurellaceae, Burkholderiaceae, Flavobacteriaceae, Corynebacteriaceae, and Staphylococcaceae were higher in the control group (Fig. 2C). The relative abundances of 15 genera were significantly different in the saliva samples of the obese group compared with that of the control samples. The relative abundances of *Prevotella*, *Granulicatella*, *Peptostreptococcaceae_XIG-1, Peptostreptococcus, Solobacterium, Mogibacterium, Catonella,* and *Peptostreptococcaceae_XIG-7* increased; meanwhile, the relative abundances of *Haemophilus*, *Lautropia, Capnocytophaga*, *Corynebacterium*, *Staphylococcus, Cardiobacterium,* and *Stenotrophomonas* decreased (Fig. 2D). Of the 15 differentially abundant genera, 13 were detected in all saliva samples. The detection frequency of *Staphylococcus* was 75.9% (22/29) in normal weight participants and 36.4% (12/33) in obese participants (*P* = 0.001). Further analysis at species levels were described in Table S8.

**Figure 3:**
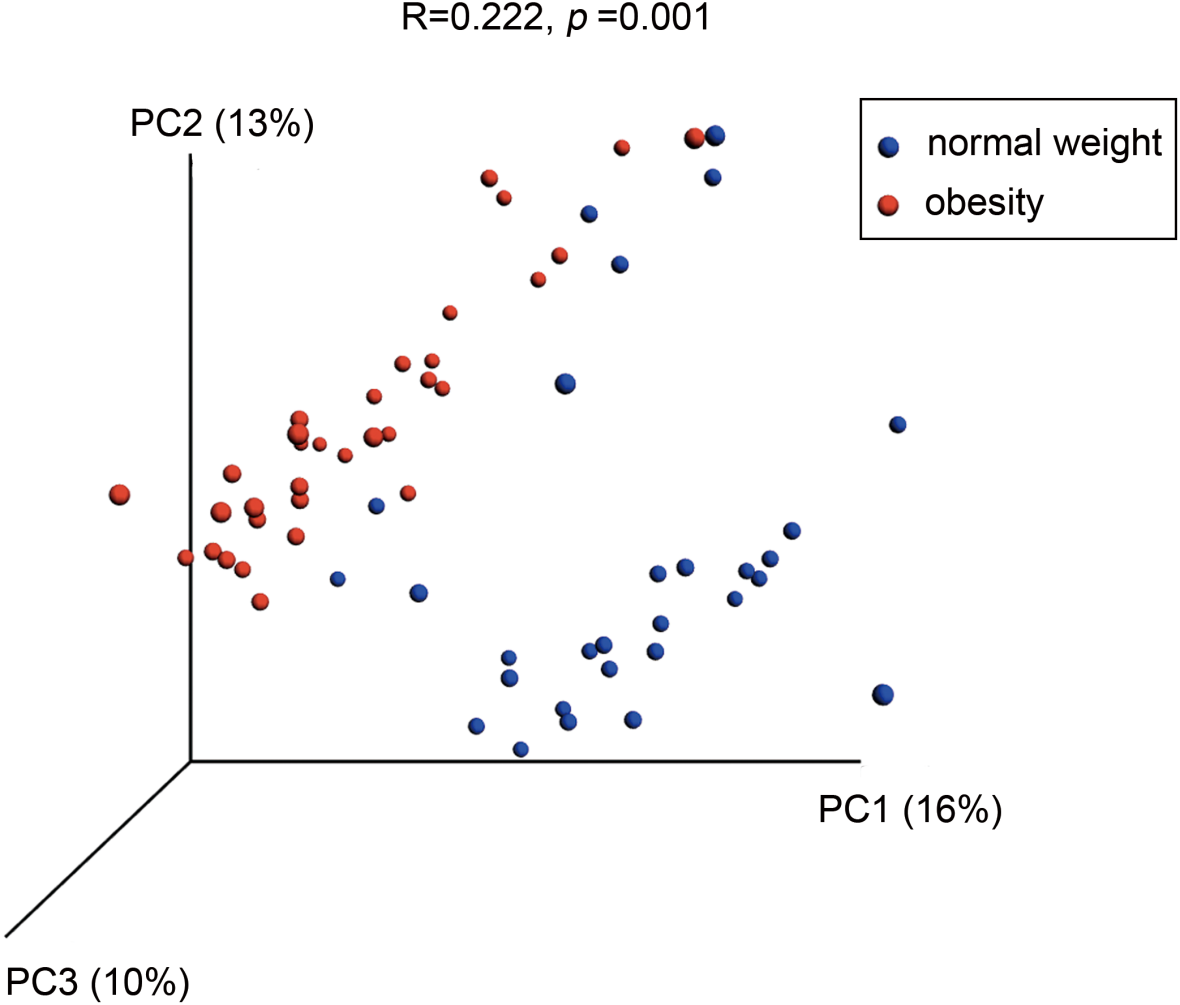
Differences in bacterial community structures of the salivary microbiome in people with obesity and normal weight controls. A principal coordinate analysis (PCoA) plot generated using unweighted UniFrac distances shows clear differences between the two groups.

A PCoA analysis of microbial OTUs revealed a difference in the microbial community composition using unweighted UniFrac distance (Fig. 3, *P* = 0.0001 by PERMANOVA), as the two groups were segregated on a PCoA plot at *P* < 0.001. The weighed version of the metric was showed in Fig. S2 (*P* = 0.0014 by PERMANOVA).

**Figure 4:**
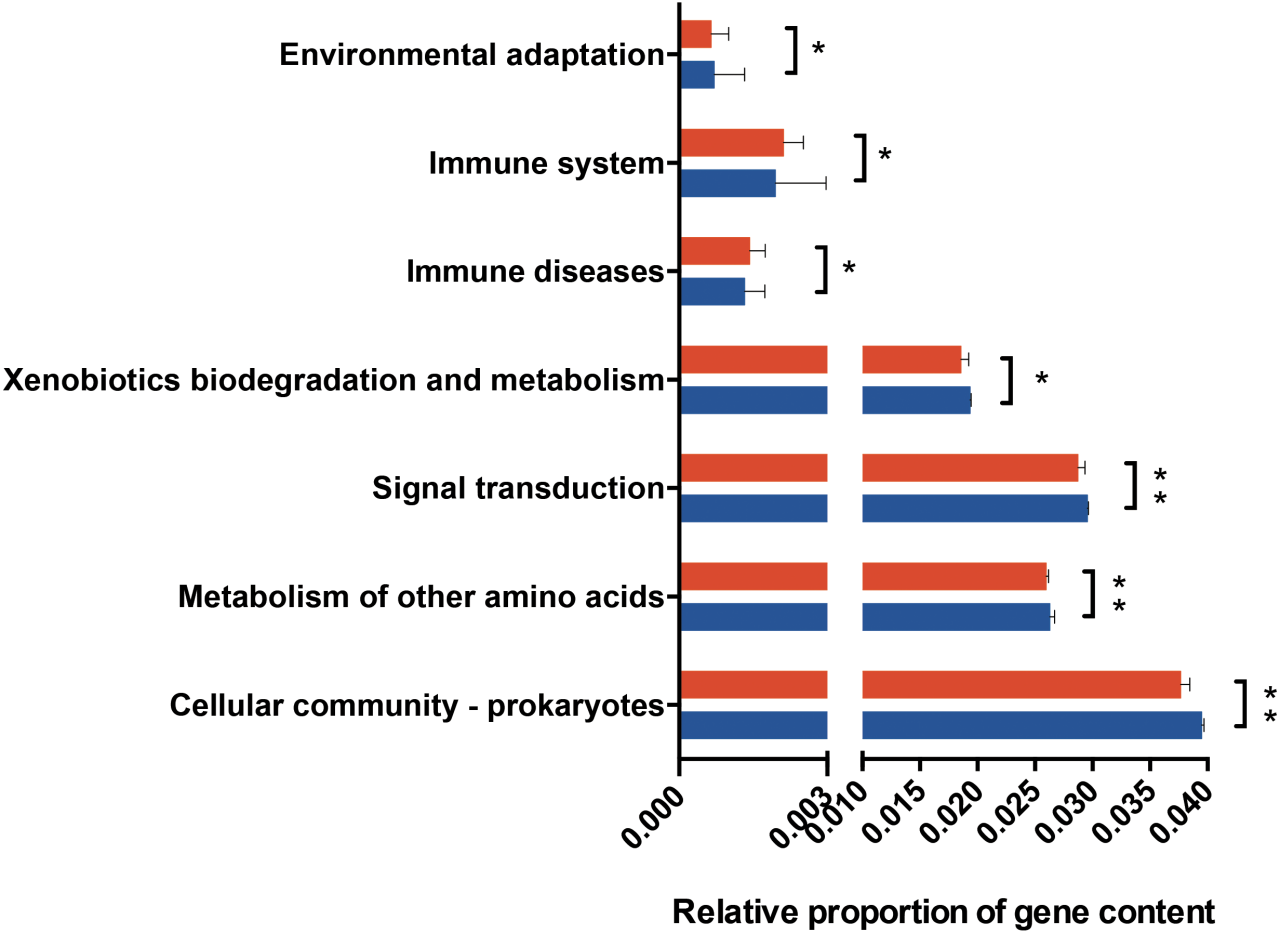
Predicted functional metagenomic changes in the saliva microbiota. Phylogenetic investigation of communities by reconstruction of unobserved states (PICRUSt) with Kegg Orthology (KO) classification was used to performed predictive function analysis in saliva samples from the two groups (level 2). Genus and KEGG pathway counts were normalized for DESeq2 size factors. The results are presented as mean ± SEM. DESeq2 in R. ^∗^*P* < 0.05; ^∗∗^*P* < 0.01. Red for obesity group, blue for normal weight group.

**Figure 5:**
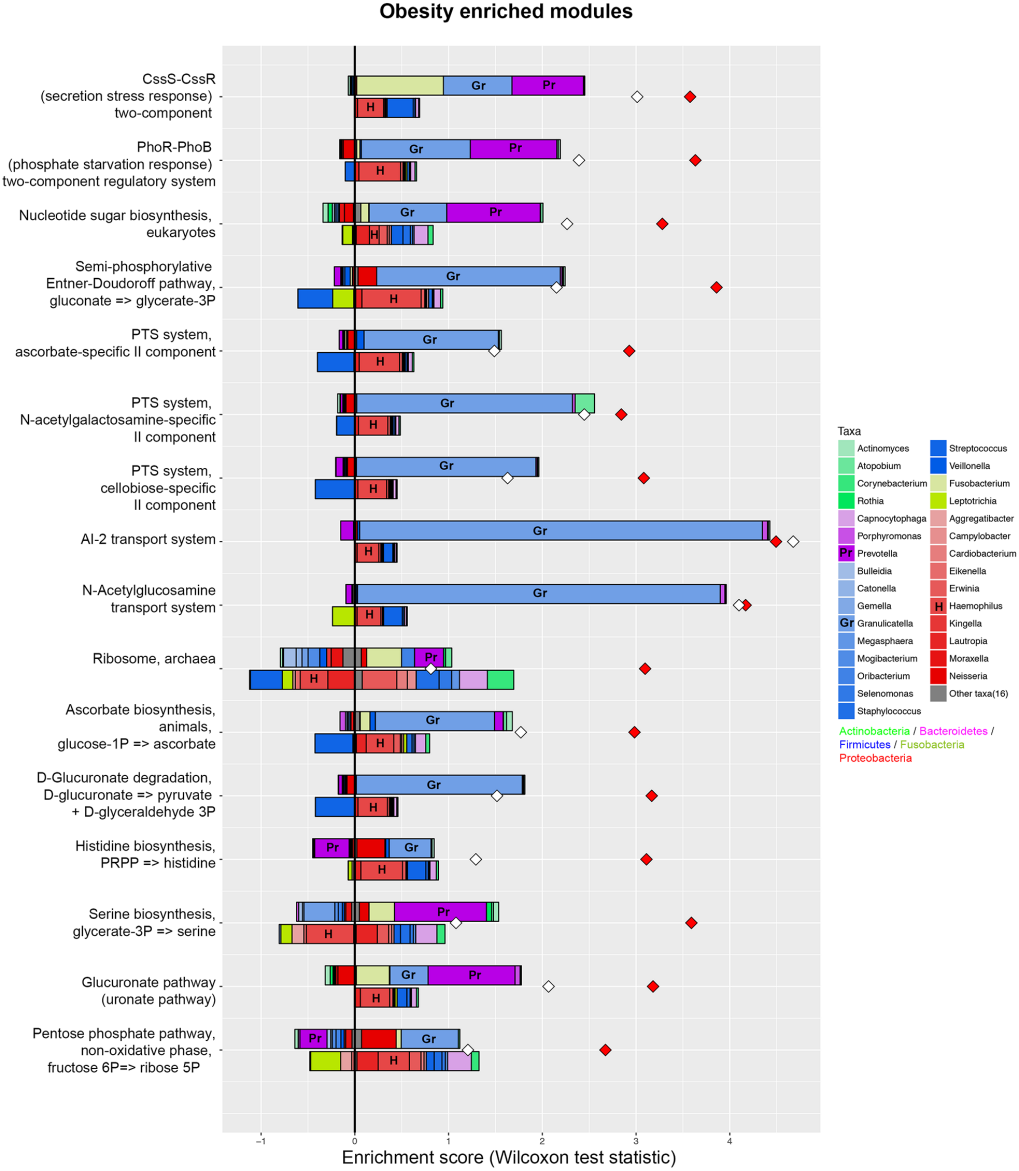
Taxonomic contributors in the obesity group determined by FishTaco. Taxon-level shift contribution profiles for several obesity-associated functional modules. For each functional module, the bar on the top-right of *Y* axis represents obese-associated genera driving the enrichment in the functional module; the bar on the top-left of *Y* axis indicates obese-associated genera attenuating functional shift; the bar on the bottom-right of *Y* axis represents genera depleted in obese people driving functional shift; the bar on the bottom-left of Y axis shows genera depleted in obese people attenuating functional shift. Red diamonds represent metagenome-based functional shift scores. White diamonds represent genus-based functional shift scores. Certain taxa are labeled by representative letters for convenience.

### Comparison of the metabolic characteristics of the salivary microbiome in people with obesity and normal weight controls

PICRUSt analysis was performed based on the 16S rRNA composition data of each sample to predict bacterial functions of members of the saliva community. Subsequent analyses revealed significant differences between the estimated functional capabilities of salivary microbiome from normal controls and the obesity groups (Fig. 4). At Level 2, eight KEGG Orthologies were found to be significantly different between salivary bacteria of obese and normal weight samples. PICRUSt analysis showed a significant increase in the microbial genetic material that is KO-annotated to immune system and immune disease. Meanwhile, the normal weight control group exhibited an increased gene involvement in environmental adaptation; Xenobiotic biodegradation and metabolism; Signal transduction; Metabolism of other amino acids; and, Cellular community—prokaryotes. In general, the salivary community associated with obesity had a stronger signature of immune disease and a decreased functional signature related to environmental adaptation and Xenobiotic biodegradation compared with the normal weight controls. Then we identified the taxa that are driving the functional shifts using FishTaco (Version 1.1.1) (Fig. 5, Table S9). We observed shifts in 16 KEGG pathway modules: Pentose phosphate pathway, non-oxidative phase, fructose 6P ⇒ ribose 5P (M00007), Glucuronate pathway (uronate pathway) (M00014), Serine biosynthesis, glycerate-3P ⇒ serine (M00020), Histidine biosynthesis, PRPP ⇒ histidine (M00026), D-Glucuronate degradation, D-glucuronate ⇒ pyruvate + D-glyceraldehyde 3P (M00061), Ascorbate biosynthesis, animals, glucose-1P ⇒ ascorbate (M00129), Ribosome, archaea (M00179), N-Acetylglucosamine transport system (M00205), AI-2 transport system (M00219), PTS system, cellobiose-specific II component (M00275), PTS system, N-acetylgalactosamine-specific II component (M00277), PTS system, ascorbate-specific II component (M00283), Semi-phosphorylative Entner-Doudoroff pathway, gluconate ⇒ glycerate-3P (M00308), Nucleotide sugar biosynthesis, eukaryotes (M00361), PhoR-PhoB (phosphate starvation response) two-component regulatory system (M00434), CssS-CssR (secretion stress response) two-component regulatory system (M00448). This analysis highlighted that *Prevotella* and *Granulicatella* were the largest contributor to alteraions in the function modules. The obese-associated genus *Granulicatella* was a major driver of the enrichment in the modules belong to carbohydrate and lipid metabolism and environmental information processing. The genus *Prevotella* was the main driver of the enrichment of glucuronate pathway and serine biosynthesis pathway modules in obese samples. In addition, in most modules the genus *Haemophilus,* which was depleted in obese people, was also a major driver of functional shifts.

## Discussion

The purpose of this study was to characterize the salivary microbiome in Chinese people with obesity, which has not been studied. In the present study, we described the distinct salivary microbial population and functional profiles of normal weight and obese people.

One effect of the microbiota is to aid the host in resisting invasion. From an ecological point of view, biodiversity is perceived to be synonymous with ecosystem health, and more diverse communities are believed to have increased stability and resistance towards invasion and other disturbances (Levine & D’Antonio, 1999). Changes in species diversity are a hallmark of many dysbiotic bacterial conditions. Decreased gut microbiome diversity has been linked to obesity (Turnbaugh et al., 2009; Le Chatelier et al., 2013), inflammatory bowel disease (IBD) (Sha et al., 2013), recurrent Clostridium difficile disease (CDAD) (Willing et al., 2010), colorectal cancer (Ahn et al., 2013), esophageal cancer (Chen et al., 2015), and Sjögren’s syndrome (Li et al., 2016). Similar associations between the altered microbial diversity and unhealthy or inflammatory states in the host have been found with the oral microbiota. Lower diversities have been found in the oral microbiomes of patients with systemic diseases, such as pediatric Crohn’s disease (Docktor et al., 2012) and hepatitis B virus-induced chronic liver disease (Ling et al., 2015). Specific to the oral ecosystem, periodontitis (Ai et al., 2017), dental caries (Simón-Soro et al., 2013) and increased candida load are associated with a decrease in microbial diversity (Kraneveld et al., 2012). It is possible that the alterations in salivary microbial diversity in people with obesity may contribute to a higher risk for oral disease.

Significant differences in the gut microbiome were identified between people with obesity and controls (Ley et al., 2006). Data obtained from animal models and human studies have revealed the correlation between obesity and altered gut phyla, despite conflicting data. In the present study, no significant differences emerged between groups at phylum level in the salivary microbiome, however, some significant variations were noted between groups at higher taxonomic levels. Salivary gram-negative *Haemphilus* and *Cardiobacterium* were elevated in the normal weight controls as compared to obesity group, which are similar to those found in a study of autoimmune rheumatoid arthritis (Zhang et al., 2015). Higher salivary *Prevotella* and lower *Lautropia*, *Corynebacterium*, and *Cardiobacterium* were also linked to esophageal cancer subjects and diabetes mellitus (Chen et al., 2015; Janem et al., 2017). These studies showed that systemic disease states correlate with relative depletion of certain salivary bacteria and microbial dysbiosis of different natures. In our study, the relative abundances of *Prevotella, Granulicatella, Peptostreptococcus, Solobacterium, Catonella*, and *Mogibacterium* were significantly higher in the obesity group. *Prevotella* species predominate in periodontal diseases and abscesses (Herrera et al., 2008), and may be involved with other bacterial species in the perpetuation of chronic periodontal and systemic inflammation (Alauzet, Marchandin & Lozniewski, 2010). It is noted that the increase abundance of *Prevotella* has been observed in several localized and systemic diseases including periodontitis, bacterial vaginosis, rheumatoid arthritis, metabolic disorders and low-grade systemic inflammation, linked to a shift towards pro-inflammatory Th helper type 17 responses (Larsen, 2017). Over-representation of Prevotellaceae is proposed as a marker of microbial dsybiosis predisposing to inflammation and metabolic disease (Furet et al., 2010). Researchers have noted that salivary *Prevotella* is positively associated with pro-inflammatory cytokine interleukin-1 beta, thus it marks the high pro-inflammatory state (Said et al., 2014; Acharya et al., 2017).

*Granulicatella* species, although considered to be a commensal member of the human oral community, have been found in endodontic infection (Siqueira & Rôças, 2006), dental abscesses (Robertson & Smith, 2009), and can also cause a variety of serious infections such as bacterial endocarditis and bacteraemia (Cargill et al., 2012). Raised salivary *Granulicatella* was also found linked to pancreatic cancer, supporting the notion that certain oral bacteria may be implicated in systemic diseases including pancreatic cancer, related to oral inflammation and confer an increased risk of systemic disease (Farrell et al., 2012). *Peptostreptococcus* species have also been associated with periodontal and endodontic infections (Riggio, Lennon & Smith, 2001); the same trend has been reported for *Mogibacterium* and *Catonella* species (Siqueira & Rôças, 2006; Robertson & Smith, 2009; Colombo et al., 2009).

A previous study failed to establish a clear correlation between obesity and the salivary microbiome maybe due to the lack of medical information (Piombino et al., 2014). Our exclusion criteria ensured that none of the participants had systemic disease, periodontal inflammation, or other oral diseases. Given the above, our study showed the altered salivary microbial diversity and composition in people with obesity, which might contribute to the risk for oral diseases such as periodontitis. Future studies using metagenomic shotgun sequencing are necessary to characterize the oral microbiome at the species level, in order to provide a better explanation.

Many studies have shown shifts in gut microbiome composition during alterations in energy balance (Turnbaugh et al., 2006) and glucose metabolism (Cani et al., 2007), as well as low-grade inflammation (Everard et al., 2011). Obesity is associated with substantial metabolic and endocrine abnormalities, including changes in sex hormone metabolism, insulin and insulin-like growth factor signaling, as well as adipokines or inflammatory pathways (Calle & Kaaks, 2004; Renehan, Zwahlen & Egger, 2015). The chronic inflammation state associated with obesity may impact the dynamic oral environment and increase the risk for oral diseases. Our findings showed that the salivary microbiome of people with obesity was significantly different with that of normal weight controls. Analysis showed dissimilarities in the relative abundance of genes involved in several KOs, which concurred with the differences in bacterial community profiles between the two groups. There was a higher signature of immune disease and a decreased functional signature related to environmental adaptation and Xenobiotics biodegradation compared with the normal weight controls.

Westcott & Schloss (2015) used two previously published datasets to analyze six *de novo* algorithms, Swarm, and the open-reference and closed-reference methods and demonstrated that *de novo* methods are the optimal method for assigning sequences into OTUs. Therefore, we used the *de novo* OTU picking method in this study. Meanwhile, we also used open reference picking methods and produce higher number of OTUs (443 OTUs) at the same filter condition. A systematic and comprehensive contrastive study is needed to compare open reference and closed reference OTU picking strategy with the one we currently used and reveal the more appropriate strategy in the future.

The strengths of our study lay in the use of strict exclusion criteria regarding periodontal disease; thus only periodontally healthy individuals were investigated. The potential influence of confounding external variables, such as pathological conditions, medications, antibiotics, and smoking, was avoided. Potential weaknesses were that we did not obtain dietary records or stool samples to investigate the correlation between the gut and oral microbiomes in people with obesity. Another limitation is that we recruited more male participants than female. While gender-related differences in microbiome and metabolome of saliva have been observed and have been associated with salivary pH and dietary protein intake (Zaura et al., 2017). The results from inference regarding metabolic profiles using PICRUSt should be interpreted conservatively for the known intra-variation in gene content. Future investigations may also benefit from larger cohorts and metagenomic shotgun sequencing, which could verify the predicted functional differences.

## Conclusions

We demonstrate that the microbial diversity, composition, and structure of the salivary microbiome of people with obesity are different from those of people of a normal weight, and provide new references that the alterations of salivary microbiome may be a systematic response to obesity, which might contribute to the risk for oral disorders. On the other hand, the expansion of some bacteria in saliva may assist the development of obesity or other systemic disorders through immune-inflammatory processes. Additionally, we predicted bacterial metabolic pathways, and found there was a higher signature of immune disease and a decreased functional signature related to environmental adaptation and Xenobiotics biodegradation in people with obesity compared with the normal weight controls. Our results offer new insights into the reciprocal impact between the salivary microbiome and obesity; however, more research is needed to explain how salivary microbiome affect the susceptibility of people with obesity to develop oral diseases and other systemic inflammatory disorders.

##  Supplemental Information

10.7717/peerj.4458/supp-1 Figure S1 The rank-abundance curve had a steep slope

10.7717/peerj.4458/supp-2 Figure S2 Differences in bacterial community structures of the salivary microbiome in people with obesity and normal weight controls (A) A PCoA plot generated using unweighted UniFrac distances (B) A PCoA plot generated using weighted UniFrac distances.

10.7717/peerj.4458/supp-3 Table S1 Summaries of pyrosequencing data for all samples OTU, operational taxonomic unit.

10.7717/peerj.4458/supp-4 Table S2 Summaries of the alpha diversity indices The alpha diversity indices Chao1, Good’s coverage, observed OTUs, Shannon index, and Phylogenetic Diversity whole tree are shown.

10.7717/peerj.4458/supp-5 Table S3 Detailed information of relative abundances of class in saliva samples summarized by group

10.7717/peerj.4458/supp-6 Table S4 Detailed information of relative abundances of oders in saliva samples summarized by group

10.7717/peerj.4458/supp-7 Table S5 Detailed information of relative abundances of families in saliva samples summarized by group

10.7717/peerj.4458/supp-8 Table S6 Detailed information of relative abundances of genera in saliva samples summarized by group

10.7717/peerj.4458/supp-9 Table S7 Detailed information of relative abundances of species in saliva samples summarized by group

10.7717/peerj.4458/supp-10 Table S8 Differentially abundant species between people with obesity and normal weight controls The relative abundances of 58 species were significantly different in the saliva samples of the obese group compared with that of the control group, in which 26 were over-represented in the obese group. (Kruskal–Wallis test, *P* < 0.05).

10.7717/peerj.4458/supp-11 Table S9 Obesity-associated functional modules in the saliva samples Eighteen obesity-associated functional modules were obtained in the saliva samples using the FishTaco software.

10.7717/peerj.4458/supp-12 Data S1 The V3–V4 hypervariable regions of the 16S rRNA gene were subjected to high-throughput sequencing by Beijing Auwigene Tech, Ltd (Beijing, China) using the Illumina Miseq PE300 sequencing platform (Illumina, Inc., CA, USA) We originally included 68 subjects, but finally kept 62. We excluded O9, O26, O27, O29, O37 and H43 for their bad periodontal status.

10.7717/peerj.4458/supp-13 Supplemental Information 1 Raw data of filtered OTU table

10.7717/peerj.4458/supp-14 Supplemental Information 2 Questionnaire An empty copy of the questionnaire we used.

## Additional Information and Declarations

### Data Availability

The following information was supplied regarding data availability: The raw data has been provided as a Supplemental File.
