# Supplementary figures and images for: Characterization of the salivary microbiome in people with obesity
Source: PeerJ. 2018 Mar 16;6:e4458. doi: 10.7717/peerj.4458 (PMC5858547; doi:10.7717/peerj.4458)

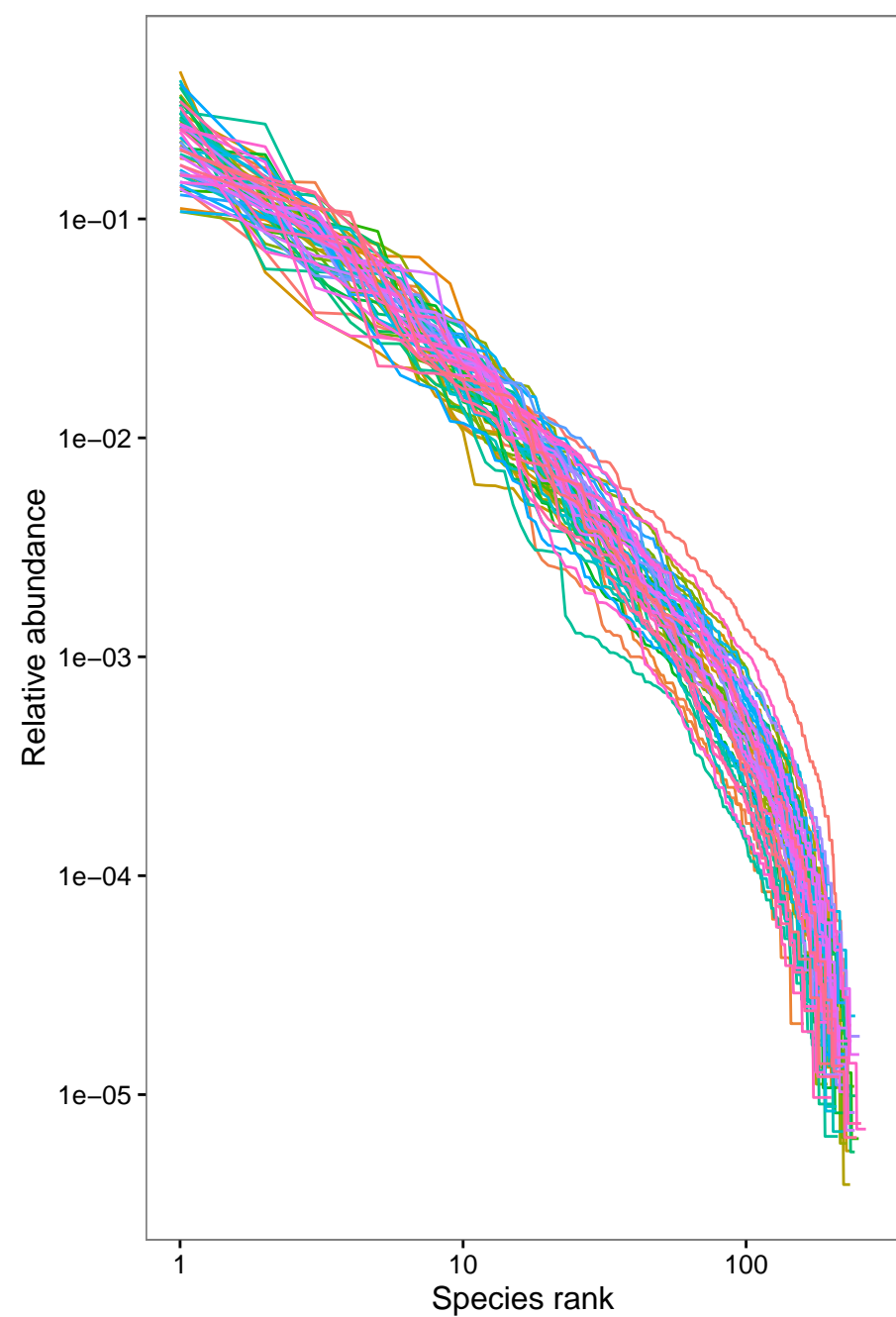

Sample

|     |     |     |     |
|-----|-----|-----|-----|
| O1  | O19 | O45 | H17 |
| O2  | O20 | H1  | H18 |
| O3  | O21 | H2  | H21 |
| O4  | O22 | H3  | H23 |
| O5  | O23 | H4  | H24 |
| O6  | O24 | H5  | H25 |
| O7  | O25 | H6  | H26 |
| O8  | O28 | H7  | H28 |
| O10 | O30 | H8  | H29 |
| O11 | O31 | H9  | H31 |
| O12 | O33 | H10 | H33 |
| O13 | O34 | H11 | H34 |
| O15 | O35 | H12 | H35 |
| O16 | O40 | H13 | H45 |
| O17 | O42 | H14 |     |
| O18 | O43 | H16 |     |

Supplement: Figure S1 [file peerj-06-4458-s001.pdf]

**A**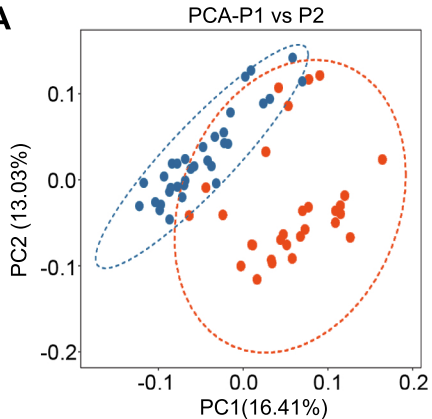**B**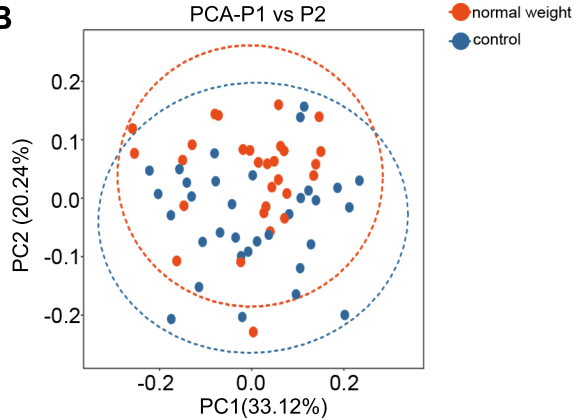

Supplement: Figure S2 — (A) A PCoA plot generated using unweighted UniFrac distances (B) A PCoA plot generated using weighted UniFrac distances. [file peerj-06-4458-s002.pdf]
