# Supplementary material for: Characterization of the salivary microbiome in people with obesity
Source: PeerJ. 2018 Mar 16;6:e4458. doi: 10.7717/peerj.4458 (PMC5858547; doi:10.7717/peerj.4458)
Supplement: Table S1 — OTU, operational taxonomic unit. [file peerj-06-4458-s003.docx]

**Table S1. Summaries of pyrosequencing data for all samples.**

| **Sample ID** | **Group** | **Raw tags** | **Clean tags** | **Final tags** | **Number of OTUs** |
| --- | --- | --- | --- | --- | --- |
| O1 | obesity | 75783 | 68986 | 48152 | 235 |
| O2 | obesity | 95971 | 83390 | 62039 | 218 |
| O3 | obesity | 104393 | 88414 | 74989 | 209 |
| O4 | obesity | 79537 | 67543 | 47388 | 160 |
| O5 | obesity | 72404 | 67545 | 35667 | 207 |
| O6 | obesity | 142424 | 119442 | 92817 | 223 |
| O7 | obesity | 97550 | 90617 | 74644 | 216 |
| O8 | obesity | 99993 | 89223 | 64607 | 217 |
| O10 | obesity | 185982 | 154282 | 126181 | 235 |
| O11 | obesity | 240590 | 214599 | 180647 | 235 |
| O12 | obesity | 94694 | 87613 | 65140 | 228 |
| O13 | obesity | 380653 | 336816 | 257477 | 233 |
| O15 | obesity | 128796 | 119554 | 89612 | 194 |
| O16 | obesity | 254920 | 233821 | 166966 | 228 |
| O17 | obesity | 99995 | 90557 | 56564 | 205 |
| O18 | obesity | 66863 | 61788 | 49291 | 211 |
| O19 | obesity | 192237 | 177352 | 156548 | 230 |
| O20 | obesity | 121999 | 113589 | 91658 | 241 |
| O21 | obesity | 143017 | 127475 | 99466 | 235 |
| O22 | obesity | 249740 | 227450 | 158807 | 250 |
| O23 | obesity | 132072 | 119788 | 93113 | 209 |
| O24 | obesity | 168385 | 154948 | 121142 | 222 |
| O25 | obesity | 85877 | 80708 | 64385 | 204 |
| O28 | obesity | 144322 | 133330 | 101241 | 245 |
| O30 | obesity | 282506 | 248828 | 182672 | 242 |
| O31 | obesity | 97435 | 93088 | 74729 | 221 |
| O33 | obesity | 152213 | 136972 | 110398 | 204 |
| O34 | obesity | 204396 | 181824 | 155100 | 211 |
| O35 | obesity | 88550 | 79080 | 59325 | 196 |
| O40 | obesity | 198655 | 181787 | 147413 | 222 |
| O42 | obesity | 151813 | 140302 | 113301 | 209 |
| O43 | obesity | 77209 | 74059 | 55496 | 215 |
| O45 | obesity | 52608 | 50206 | 35798 | 197 |
| H1 | normal weight | 156691 | 138675 | 118860 | 208 |
| H2 | normal weight | 62822 | 61597 | 43692 | 243 |
| H3 | normal weight | 82083 | 79953 | 58860 | 217 |
| H4 | normal weight | 102580 | 98996 | 67278 | 219 |
| H5 | normal weight | 103035 | 100862 | 74464 | 224 |
| H6 | normal weight | 94196 | 92704 | 65268 | 223 |
| H7 | normal weight | 80788 | 80084 | 65600 | 219 |
| H8 | normal weight | 62603 | 61517 | 38082 | 232 |
| H9 | normal weight | 56224 | 55562 | 35101 | 204 |
| H10 | normal weight | 144233 | 139564 | 90382 | 230 |
| H11 | normal weight | 69953 | 68657 | 51088 | 216 |
| H12 | normal weight | 85808 | 83432 | 54116 | 252 |
| H13 | normal weight | 111756 | 109247 | 81058 | 220 |
| H14 | normal weight | 165697 | 160833 | 120741 | 242 |
| H16 | normal weight | 187050 | 181168 | 145382 | 242 |
| H17 | normal weight | 70323 | 68808 | 49614 | 226 |
| H18 | normal weight | 135333 | 132993 | 97150 | 232 |
| H21 | normal weight | 88583 | 86703 | 65561 | 251 |
| H23 | normal weight | 88322 | 86650 | 56870 | 236 |
| H24 | normal weight | 63468 | 62178 | 46712 | 196 |
| H25 | normal weight | 57634 | 56727 | 41588 | 208 |
| H26 | normal weight | 92398 | 89182 | 79097 | 190 |
| H28 | normal weight | 186093 | 171232 | 143659 | 265 |
| H29 | normal weight | 130072 | 116148 | 103035 | 200 |
| H31 | normal weight | 174494 | 158783 | 135344 | 255 |
| H33 | normal weight | 104127 | 94545 | 82854 | 213 |
| H34 | normal weight | 205457 | 186767 | 156905 | 246 |
| H35 | normal weight | 97012 | 89909 | 73954 | 220 |
| H45 | normal weight | 146204 | 134090 | 72221 | 198 |
