# Supplementary material for: Characterization of the salivary microbiome in people with obesity
Source: PeerJ. 2018 Mar 16;6:e4458. doi: 10.7717/peerj.4458 (PMC5858547; doi:10.7717/peerj.4458)
Supplement: Table S2 — The alpha diversity indices Chao1, Good’s coverage, observed OTUs, Shannon index, and Phylogenetic Diversity whole tree are shown. [file peerj-06-4458-s004.docx]

**Table S2. Summaries of the alpha diversity indices.**

| SampleID | chao1 | goods_coverage | observed_species | PD_whole_tree | shannon |
| --- | --- | --- | --- | --- | --- |
| H1 | 213.9375 | 0.999230769 | 192 | 13.55458 | 4.335194765 |
| H2 | 255.4375 | 0.999373219 | 241 | 16.11699 | 5.042064805 |
| H3 | 219.4 | 0.999373219 | 204 | 13.94526 | 4.417093096 |
| H4 | 225.6470588 | 0.999287749 | 208 | 14.16113 | 5.103606789 |
| H5 | 252.3333333 | 0.999088319 | 211 | 14.58984 | 5.136839669 |
| H6 | 215 | 0.99954416 | 205 | 14.84834 | 5.212634093 |
| H7 | 244.1538462 | 0.999088319 | 206 | 14.6654 | 3.529742046 |
| H8 | 249.0909091 | 0.999401709 | 230 | 15.64706 | 5.33422075 |
| H9 | 249.1111111 | 0.999173789 | 204 | 14.77288 | 5.176377824 |
| H10 | 261.6666667 | 0.999059829 | 203 | 13.77548 | 5.008690981 |
| H11 | 216.125 | 0.999458689 | 209 | 14.39814 | 4.919386885 |
| H12 | 260 | 0.999316239 | 237 | 15.24439 | 5.171722203 |
| H13 | 268.9090909 | 0.998860399 | 198 | 12.95431 | 4.828804932 |
| H14 | 222.5555556 | 0.999430199 | 212 | 14.00878 | 4.489784563 |
| H16 | 218 | 0.999401709 | 208 | 14.48145 | 4.665359384 |
| H17 | 233.5454545 | 0.999458689 | 218 | 14.84835 | 5.209476618 |
| H18 | 242.6 | 0.999316239 | 215 | 14.33096 | 4.898026414 |
| H21 | 245.5357143 | 0.999230769 | 233 | 15.36696 | 4.933712641 |
| H23 | 253.2727273 | 0.998945869 | 223 | 14.63133 | 4.410469693 |
| H24 | 219.875 | 0.999373219 | 191 | 13.24834 | 5.002223426 |
| H25 | 220.0714286 | 0.999344729 | 202 | 13.89149 | 5.008849767 |
| H26 | 199.4736842 | 0.999230769 | 181 | 12.33177 | 3.852474455 |
| H28 | 249.0714286 | 0.999344729 | 231 | 15.84407 | 5.478484674 |
| H29 | 198.5882353 | 0.999145299 | 173 | 13.20782 | 4.445996232 |
| H31 | 238.75 | 0.998945869 | 211 | 13.69385 | 4.359871598 |
| H33 | 198.5 | 0.999230769 | 179 | 12.6013 | 3.790943124 |
| H34 | 248.4615385 | 0.998974359 | 200 | 13.5406 | 4.276384011 |
| H35 | 230.4 | 0.999230769 | 207 | 15.01204 | 4.756939552 |
| H45 | 192 | 0.999316239 | 180 | 13.83667 | 4.4554891 |
| O1 | 239.6666667 | 0.99962963 | 231 | 15.62287 | 5.984157164 |
| O2 | 225.5882353 | 0.999373219 | 212 | 14.88279 | 4.478823533 |
| O3 | 221.75 | 0.999002849 | 192 | 14.39183 | 3.752118153 |
| O4 | 167.2142857 | 0.999458689 | 155 | 11.7348 | 4.120472043 |
| O5 | 228 | 0.999401709 | 207 | 15.39506 | 4.887404561 |
| O6 | 251.8 | 0.999059829 | 199 | 13.76706 | 4.863938737 |
| O7 | 212.7142857 | 0.999316239 | 193 | 14.20643 | 4.643944368 |
| O8 | 212 | 0.99974359 | 208 | 14.20244 | 4.178371067 |
| O10 | 222.6521739 | 0.999487179 | 216 | 15.14087 | 4.268317022 |
| O11 | 228.4 | 0.999373219 | 213 | 14.6708 | 4.242276742 |
| O12 | 228.9285714 | 0.999487179 | 218 | 15.34746 | 4.81558828 |
| O13 | 208.7692308 | 0.99974359 | 206 | 14.94381 | 5.392767494 |
| O15 | 185.5 | 0.999487179 | 177 | 13.51652 | 4.365454242 |
| O16 | 202.875 | 0.99962963 | 198 | 14.87062 | 3.998328958 |
| O17 | 198 | 0.99954416 | 188 | 14.3903 | 4.909460299 |
| O18 | 226.4285714 | 0.999287749 | 205 | 13.521 | 5.110792464 |
| O19 | 211.2 | 0.99962963 | 206 | 14.18577 | 4.721491245 |
| O20 | 227.5454545 | 0.999458689 | 212 | 14.6042 | 4.044014065 |
| O21 | 226.1578947 | 0.999373219 | 214 | 14.93623 | 4.520775177 |
| O22 | 234.1764706 | 0.999430199 | 223 | 15.76211 | 4.795013257 |
| O23 | 198.5454545 | 0.999316239 | 186 | 13.18067 | 4.177353497 |
| O24 | 206 | 0.999487179 | 197 | 14.14037 | 4.289516319 |
| O25 | 228.5 | 0.999259259 | 196 | 14.93454 | 4.428802758 |
| O28 | 242.8947368 | 0.999145299 | 220 | 15.27531 | 4.22387099 |
| O30 | 242.2307692 | 0.999316239 | 221 | 15.38528 | 4.615532926 |
| O31 | 226.2307692 | 0.999316239 | 205 | 14.89338 | 4.240765849 |
| O33 | 213.4615385 | 0.999145299 | 180 | 12.73791 | 4.234642432 |
| O34 | 184.75 | 0.999202279 | 169 | 12.00669 | 3.37157341 |
| O35 | 226.2727273 | 0.999116809 | 184 | 13.16486 | 4.380520144 |
| O40 | 227 | 0.998974359 | 185 | 13.65867 | 4.131204004 |
| O42 | 207.3333333 | 0.999059829 | 178 | 13.17117 | 3.684776994 |
| O43 | 220.25 | 0.999458689 | 206 | 14.81242 | 5.178591318 |
| O45 | 215.8947368 | 0.999202279 | 196 | 14.17146 | 4.181413665 |
