# Supplementary material for: Characterization of the salivary microbiome in people with obesity
Source: PeerJ. 2018 Mar 16;6:e4458. doi: 10.7717/peerj.4458 (PMC5858547; doi:10.7717/peerj.4458)
Supplement: Table S3 [file peerj-06-4458-s005.docx]

| OTU | Test-Statistic | P | normal_weight_mean | obesity_mean |
| --- | --- | --- | --- | --- |
| k__Bacteria;p__Firmicutes;c__Erysipelotrichia | 15.5486391 | 8.04E-05 | 0.000755201 | 0.001506545 |
| k__Bacteria;p__Proteobacteria;c__Gammaproteobacteria | 11.70448326 | 0.000623497 | 0.164132749 | 0.108902758 |
| k__Bacteria;p__Bacteroidetes;c__Bacteroidia | 8.242474001 | 0.004092132 | 0.139670933 | 0.196114587 |
| k__Bacteria;p__Bacteroidetes;c__Flavobacteriia | 7.529531771 | 0.006069568 | 0.012748904 | 0.008017513 |
| k__Bacteria;p__Firmicutes;c__Mollicutes | 4.641428703 | 0.03120901 | 0.000126696 | 0.000143447 |
| k__Bacteria;p__Firmicutes;c__Clostridia | 4.389162562 | 0.036168074 | 0.024728377 | 0.027698937 |
| k__Bacteria;p__Gracilibacteria_GN02;c__GN02_C-2 | 2.806556057 | 0.093879719 | 0.001390592 | 0.000431518 |
| k__Bacteria;p__Fusobacteria;c__Fusobacteriia | 2.655172414 | 0.103213565 | 0.033749585 | 0.024704643 |
| k__Bacteria;p__Bacteroidetes;c__Bacteroidetes_C-1 | 1.602412008 | 0.205561729 | 0.000713043 | 9.79E-05 |
| k__Bacteria;p__SR1;c__SR1_C-1 | 1.438061789 | 0.230453243 | 0.003233288 | 0.00781292 |
| k__Bacteria;p__Saccharibacteria_TM7;c__TM7_C-1 | 1.134547445 | 0.286807435 | 0.006564494 | 0.00607076 |
| k__Bacteria;p__Synergistetes;c__Synergistia | 0.886566202 | 0.346409534 | 0.000180258 | 0.000153232 |
| k__Bacteria;p__Spirochaetes;c__Spirochaetia | 0.854338822 | 0.355327797 | 0.001416435 | 0.000897701 |
| k__Bacteria;p__Gracilibacteria_GN02;c__GN02_C-1 | 0.775048563 | 0.378659756 | 0.000205497 | 0.000167368 |
| k__Bacteria;p__Actinobacteria;c__Actinobacteria | 0.507588197 | 0.476184775 | 0.054098972 | 0.04719977 |
| k__Bacteria;p__Actinobacteria;c__Coriobacteriia | 0.41205155 | 0.520930052 | 0.004381885 | 0.003134205 |
| k__Bacteria;p__Firmicutes;c__Negativicutes | 0.295019157 | 0.587021944 | 0.038145215 | 0.039725049 |
| k__Bacteria;p__Proteobacteria;c__Betaproteobacteria | 0.250833458 | 0.616488825 | 0.184312476 | 0.206737089 |
| k__Bacteria;p__Firmicutes;c__Bacilli | 0.047818082 | 0.826904424 | 0.326204366 | 0.317124645 |
| k__Bacteria;p__Proteobacteria;c__Epsilonproteobacteria | 0.014380256 | 0.90454834 | 0.003241035 | 0.003359432 |
