# Supplementary material for: Characterization of the salivary microbiome in people with obesity
Source: PeerJ. 2018 Mar 16;6:e4458. doi: 10.7717/peerj.4458 (PMC5858547; doi:10.7717/peerj.4458)
Supplement: Table S4 [file peerj-06-4458-s006.docx]

| OTU | Test-Statistic | P | normal_weight_mean | obesity_mean |
| --- | --- | --- | --- | --- |
| k__Bacteria;p__Actinobacteria;c__Actinobacteria;o__Corynebacteriales | 15.88421157 | 6.73E-05 | 0.003542379 | 0.001453431 |
| k__Bacteria;p__Firmicutes;c__Erysipelotrichia;o__Erysipelotrichales | 15.5486391 | 8.04E-05 | 0.000755201 | 0.001506545 |
| k__Bacteria;p__Proteobacteria;c__Betaproteobacteria;o__Burkholderiales | 11.51221575 | 0.000691403 | 0.00740239 | 0.002293258 |
| k__Bacteria;p__Proteobacteria;c__Gammaproteobacteria;o__Pasteurellales | 11.32154053 | 0.000766131 | 0.161342883 | 0.104695465 |
| k__Bacteria;p__Proteobacteria;c__Gammaproteobacteria;o__Cardiobacteriales | 10.48320645 | 0.001204645 | 0.001264114 | 0.000427185 |
| k__Bacteria;p__Bacteroidetes;c__Bacteroidia;o__Bacteroidales | 8.242474001 | 0.004092132 | 0.139670933 | 0.196114587 |
| k__Bacteria;p__Bacteroidetes;c__Flavobacteriia;o__Flavobacteriales | 7.529531771 | 0.006069568 | 0.012748904 | 0.008017513 |
| k__Bacteria;p__Proteobacteria;c__Gammaproteobacteria;o__Xanthomonadales | 6.775190327 | 0.009243363 | 0.000307566 | 0.000236785 |
| k__Bacteria;p__Firmicutes;c__Mollicutes;o__Mycoplasmatales | 4.641428703 | 0.03120901 | 0.000126696 | 0.000143447 |
| k__Bacteria;p__Firmicutes;c__Clostridia;o__Clostridiales | 4.389162562 | 0.036168074 | 0.024728377 | 0.027698937 |
| k__Bacteria;p__Firmicutes;c__Bacilli;o__Bacillales | 3.035726725 | 0.08145007 | 0.039749561 | 0.030844149 |
| k__Bacteria;p__Gracilibacteria_GN02;c__GN02_C-2;o__GN02_O-2 | 2.806556057 | 0.093879719 | 0.001390592 | 0.000431518 |
| k__Bacteria;p__Fusobacteria;c__Fusobacteriia;o__Fusobacteriales | 2.655172414 | 0.103213565 | 0.033749585 | 0.024704643 |
| k__Bacteria;p__Bacteroidetes;c__Bacteroidetes_C-1;o__Bacteroidetes_O-1 | 1.602412008 | 0.205561729 | 0.000713043 | 9.79E-05 |
| k__Bacteria;p__SR1;c__SR1_C-1;o__SR1_O-1 | 1.438061789 | 0.230453243 | 0.003233288 | 0.00781292 |
| k__Bacteria;p__Saccharibacteria_TM7;c__TM7_C-1;o__TM7_O-1 | 1.134547445 | 0.286807435 | 0.006564494 | 0.00607076 |
| k__Bacteria;p__Synergistetes;c__Synergistia;o__Synergistales | 0.886566202 | 0.346409534 | 0.000180258 | 0.000153232 |
| k__Bacteria;p__Spirochaetes;c__Spirochaetia;o__Spirochaetales | 0.854338822 | 0.355327797 | 0.001416435 | 0.000897701 |
| k__Bacteria;p__Gracilibacteria_GN02;c__GN02_C-1;o__GN02_O-1 | 0.775048563 | 0.378659756 | 0.000205497 | 0.000167368 |
| k__Bacteria;p__Proteobacteria;c__Betaproteobacteria;o__Neisseriales | 0.46817933 | 0.493825956 | 0.176910086 | 0.204443831 |
| k__Bacteria;p__Actinobacteria;c__Coriobacteriia;o__Coriobacteriales | 0.41205155 | 0.520930052 | 0.004381885 | 0.003134205 |
| k__Bacteria;p__Proteobacteria;c__Gammaproteobacteria;o__Pseudomonadales | 0.310543862 | 0.577346641 | 0.000950997 | 0.0032341 |
| k__Bacteria;p__Firmicutes;c__Negativicutes;o__Selenomonadales | 0.295019157 | 0.587021944 | 0.038145215 | 0.039725049 |
| k__Bacteria;p__Actinobacteria;c__Actinobacteria;o__Actinomycetales | 0.210230383 | 0.646586892 | 0.050556593 | 0.045746339 |
| k__Bacteria;p__Proteobacteria;c__Gammaproteobacteria;o__Enterobacteriales | 0.024134247 | 0.876543815 | 0.000267189 | 0.000309223 |
| k__Bacteria;p__Proteobacteria;c__Epsilonproteobacteria;o__Campylobacterales | 0.014380256 | 0.90454834 | 0.003241035 | 0.003359432 |
| k__Bacteria;p__Firmicutes;c__Bacilli;o__Lactobacillales | 0.006020799 | 0.938151168 | 0.286454805 | 0.286280496 |
