# Supplementary material for: Characterization of the salivary microbiome in people with obesity
Source: PeerJ. 2018 Mar 16;6:e4458. doi: 10.7717/peerj.4458 (PMC5858547; doi:10.7717/peerj.4458)
Supplement: Table S5 [file peerj-06-4458-s007.docx]

| OTU | Test-Statistic | P | normal_weight_mean | obesity_mean |
| --- | --- | --- | --- | --- |
| k__Bacteria;p__Actinobacteria;c__Actinobacteria;o__Corynebacteriales;f__Corynebacteriaceae | 15.88421157 | 6.73E-05 | 0.003542379 | 0.001453431 |
| k__Bacteria;p__Firmicutes;c__Erysipelotrichia;o__Erysipelotrichales;f__Erysipelotrichaceae | 15.5486391 | 8.04E-05 | 0.000755201 | 0.001506545 |
| k__Bacteria;p__Proteobacteria;c__Betaproteobacteria;o__Burkholderiales;f__Burkholderiaceae | 12.09379509 | 0.000505899 | 0.006915191 | 0.001910146 |
| k__Bacteria;p__Proteobacteria;c__Gammaproteobacteria;o__Pasteurellales;f__Pasteurellaceae | 11.32154053 | 0.000766131 | 0.161342883 | 0.104695465 |
| k__Bacteria;p__Proteobacteria;c__Gammaproteobacteria;o__Cardiobacteriales;f__Cardiobacteriaceae | 10.48320645 | 0.001204645 | 0.001264114 | 0.000427185 |
| k__Bacteria;p__Firmicutes;c__Bacilli;o__Bacillales;f__Staphylococcaceae | 9.851939004 | 0.001696528 | 0.001752189 | 4.22E-05 |
| k__Bacteria;p__Bacteroidetes;c__Bacteroidia;o__Bacteroidales;f__Prevotellaceae | 9.329203364 | 0.002255304 | 0.078789421 | 0.127020967 |
| k__Bacteria;p__Firmicutes;c__Clostridia;o__Clostridiales;f__Peptostreptococcaceae_XI | 8.903269145 | 0.002846606 | 0.008448184 | 0.011817362 |
| k__Bacteria;p__Firmicutes;c__Bacilli;o__Lactobacillales;f__Carnobacteriaceae | 7.842414291 | 0.00510344 | 0.016910846 | 0.030160692 |
| k__Bacteria;p__Bacteroidetes;c__Flavobacteriia;o__Flavobacteriales;f__Flavobacteriaceae | 7.529531771 | 0.006069568 | 0.012748904 | 0.008017513 |
| k__Bacteria;p__Proteobacteria;c__Gammaproteobacteria;o__Xanthomonadales;f__Xanthomonadaceae | 6.775190327 | 0.009243363 | 0.000307566 | 0.000236785 |
| k__Bacteria;p__Proteobacteria;c__Betaproteobacteria;o__Burkholderiales;f__Comamonadaceae | 6.484599692 | 0.010881304 | 0.000487199 | 0.000383112 |
| k__Bacteria;p__Firmicutes;c__Clostridia;o__Clostridiales;f__Peptococcaceae | 5.85420524 | 0.015539874 | 0.000183946 | 0.000350759 |
| k__Bacteria;p__Fusobacteria;c__Fusobacteriia;o__Fusobacteriales;f__Leptotrichiaceae | 4.689704931 | 0.030343858 | 0.009282957 | 0.006843501 |
| k__Bacteria;p__Firmicutes;c__Mollicutes;o__Mycoplasmatales;f__Mycoplasmataceae | 4.641428703 | 0.03120901 | 0.000126696 | 0.000143447 |
| k__Bacteria;p__Firmicutes;c__Clostridia;o__Clostridiales;f__Peptoniphilaceae | 3.035726725 | 0.08145007 | 0.000670291 | 0.000753269 |
| k__Bacteria;p__Gracilibacteria_GN02;c__GN02_C-2;o__GN02_O-2;f__GN02_F-2 | 2.806556057 | 0.093879719 | 0.001390592 | 0.000431518 |
| k__Bacteria;p__Firmicutes;c__Bacilli;o__Bacillales;f__Gemellaceae | 2.091108126 | 0.14815857 | 0.037997372 | 0.030801959 |
| k__Bacteria;p__Bacteroidetes;c__Bacteroidia;o__Bacteroidales;f__Porphyromonadaceae | 1.630143803 | 0.20168316 | 0.060616326 | 0.068627689 |
| k__Bacteria;p__Bacteroidetes;c__Bacteroidetes_C-1;o__Bacteroidetes_O-1;f__Bacteroidetes_F-1 | 1.602412008 | 0.205561729 | 0.000713043 | 9.79E-05 |
| k__Bacteria;p__SR1;c__SR1_C-1;o__SR1_O-1;f__SR1_F-1 | 1.438061789 | 0.230453243 | 0.003233288 | 0.00781292 |
| k__Bacteria;p__Firmicutes;c__Clostridia;o__Clostridiales;f__Lachnospiraceae_XIV | 1.421157387 | 0.233212997 | 0.014062373 | 0.013002914 |
| k__Bacteria;p__Saccharibacteria_TM7;c__TM7_C-1;o__TM7_O-1;f__TM7_F-1 | 1.134547445 | 0.286807435 | 0.006564494 | 0.00607076 |
| k__Bacteria;p__Synergistetes;c__Synergistia;o__Synergistales;f__Synergistaceae | 0.886566202 | 0.346409534 | 0.000180258 | 0.000153232 |
| k__Bacteria;p__Spirochaetes;c__Spirochaetia;o__Spirochaetales;f__Spirochaetaceae | 0.854338822 | 0.355327797 | 0.001416435 | 0.000897701 |
| k__Bacteria;p__Fusobacteria;c__Fusobacteriia;o__Fusobacteriales;f__Fusobacteriaceae | 0.853908544 | 0.355448976 | 0.024466628 | 0.017861142 |
| k__Bacteria;p__Gracilibacteria_GN02;c__GN02_C-1;o__GN02_O-1;f__GN02_F-1 | 0.775048563 | 0.378659756 | 0.000205497 | 0.000167368 |
| k__Bacteria;p__Bacteroidetes;c__Bacteroidia;o__Bacteroidales;f__Bacteroidales_F-2 | 0.468415242 | 0.493717136 | 0.000265185 | 0.000465931 |
| k__Bacteria;p__Proteobacteria;c__Betaproteobacteria;o__Neisseriales;f__Neisseriaceae | 0.46817933 | 0.493825956 | 0.176910086 | 0.204443831 |
| k__Bacteria;p__Actinobacteria;c__Coriobacteriia;o__Coriobacteriales;f__Coriobacteriaceae | 0.41205155 | 0.520930052 | 0.004381885 | 0.003134205 |
| k__Bacteria;p__Firmicutes;c__Clostridia;o__Clostridiales;f__Ruminococcaceae | 0.41205155 | 0.520930052 | 0.001363585 | 0.001774632 |
| k__Bacteria;p__Proteobacteria;c__Gammaproteobacteria;o__Pseudomonadales;f__Moraxellaceae | 0.310543862 | 0.577346641 | 0.000950997 | 0.0032341 |
| k__Bacteria;p__Firmicutes;c__Negativicutes;o__Selenomonadales;f__Veillonellaceae | 0.295019157 | 0.587021944 | 0.038145215 | 0.039725049 |
| k__Bacteria;p__Firmicutes;c__Bacilli;o__Lactobacillales;f__Streptococcaceae | 0.150519978 | 0.698038943 | 0.266879269 | 0.254003622 |
| k__Bacteria;p__Actinobacteria;c__Actinobacteria;o__Actinomycetales;f__Micrococcaceae | 0.026322337 | 0.871115602 | 0.028616374 | 0.029106959 |
| k__Bacteria;p__Proteobacteria;c__Gammaproteobacteria;o__Enterobacteriales;f__Enterobacteriaceae | 0.024134247 | 0.876543815 | 0.000267189 | 0.000309223 |
| k__Bacteria;p__Proteobacteria;c__Epsilonproteobacteria;o__Campylobacterales;f__Campylobacteraceae | 0.014380256 | 0.90454834 | 0.003241035 | 0.003359432 |
| k__Bacteria;p__Firmicutes;c__Bacilli;o__Lactobacillales;f__Aerococcaceae | 0.006029302 | 0.9381076 | 0.00266469 | 0.002116182 |
| k__Bacteria;p__Actinobacteria;c__Actinobacteria;o__Actinomycetales;f__Actinomycetaceae | 0.004030452 | 0.949379632 | 0.021940219 | 0.01663938 |
