# Supplementary material for: Characterization of the salivary microbiome in people with obesity
Source: PeerJ. 2018 Mar 16;6:e4458. doi: 10.7717/peerj.4458 (PMC5858547; doi:10.7717/peerj.4458)
Supplement: Table S6 [file peerj-06-4458-s008.docx]

| OTU | Test-Statistic | P | normal_weight_mean | obesity_mean |
| --- | --- | --- | --- | --- |
| k__Bacteria;p__Bacteroidetes;c__Bacteroidia;o__Bacteroidales;f__Prevotellaceae;g__Prevotella | 7.921630094 | 0.004884724 | 0.068121285 | 0.10778095 |
| k__Bacteria;p__Firmicutes;c__Bacilli;o__Lactobacillales;f__Carnobacteriaceae;g__Granulicatella | 7.842414291 | 0.00510344 | 0.016910846 | 0.030160692 |
| k__Bacteria;p__Bacteroidetes;c__Bacteroidia;o__Bacteroidales;f__Prevotellaceae;g__Alloprevotella | 3.873264666 | 0.049061003 | 0.010668136 | 0.019240016 |
| k__Bacteria;p__Firmicutes;c__Clostridia;o__Clostridiales;f__Peptostreptococcaceae_XI;g__Peptostreptococcaceae_XIG-1 | 7.147385182 | 0.007507336 | 0.00241188 | 0.004172127 |
| k__Bacteria;p__Proteobacteria;c__Betaproteobacteria;o__Neisseriales;f__Neisseriaceae;Other | 4.577977493 | 0.032385418 | 0.000436099 | 0.002079136 |
| k__Bacteria;p__Firmicutes;c__Clostridia;o__Clostridiales;f__Peptostreptococcaceae_XI;g__Peptostreptococcus | 9.243220381 | 0.002363689 | 0.001251882 | 0.002522018 |
| k__Bacteria;p__Firmicutes;c__Erysipelotrichia;o__Erysipelotrichales;f__Erysipelotrichaceae;g__Solobacterium | 15.5486391 | 8.04E-05 | 0.000755201 | 0.001506545 |
| k__Bacteria;p__Firmicutes;c__Clostridia;o__Clostridiales;f__Peptostreptococcaceae_XI;g__Mogibacterium | 11.03851321 | 0.000892384 | 0.000972043 | 0.001665186 |
| k__Bacteria;p__Firmicutes;c__Clostridia;o__Clostridiales;f__Lachnospiraceae_XIV;g__Lachnoanaerobaculum | 4.330248296 | 0.037440784 | 0.001327284 | 0.001931853 |
| k__Bacteria;p__Firmicutes;c__Clostridia;o__Clostridiales;f__Lachnospiraceae_XIV;g__Catonella | 8.405284371 | 0.003741319 | 0.001021683 | 0.001564876 |
| k__Bacteria;p__Firmicutes;c__Clostridia;o__Clostridiales;f__Peptostreptococcaceae_XI;g__Peptostreptococcaceae_XIG-7 | 6.629766672 | 0.01002884 | 0.000386009 | 0.000810139 |
| k__Bacteria;p__Saccharibacteria_TM7;c__TM7_C-1;o__TM7_O-1;f__TM7_F-1;g__TM7_G-3 | 3.873264666 | 0.049061003 | 0.000857292 | 0.001186628 |
| k__Bacteria;p__Fusobacteria;c__Fusobacteriia;o__Fusobacteriales;f__Leptotrichiaceae;Other | 9.047599022 | 0.0026304 | 3.15E-05 | 0.000359819 |
| k__Bacteria;p__Firmicutes;c__Clostridia;o__Clostridiales;f__Peptococcaceae;g__Peptococcus | 5.85420524 | 0.015539874 | 0.000183946 | 0.000350759 |
| k__Bacteria;p__Firmicutes;c__Mollicutes;o__Mycoplasmatales;f__Mycoplasmataceae;g__Mycoplasma | 4.641428703 | 0.03120901 | 0.000126696 | 0.000143447 |
| k__Bacteria;p__Proteobacteria;c__Gammaproteobacteria;o__Pseudomonadales;f__Moraxellaceae;g__Acinetobacter | 4.155893915 | 0.041489868 | 0.000295862 | 0.00027077 |
| k__Bacteria;p__Proteobacteria;c__Betaproteobacteria;o__Burkholderiales;f__Comamonadaceae;g__Delftia | 5.000199035 | 0.025344404 | 0.000273061 | 0.000221118 |
| k__Bacteria;p__Proteobacteria;c__Gammaproteobacteria;o__Xanthomonadales;f__Xanthomonadaceae;g__Stenotrophomonas | 6.775190327 | 0.009243363 | 0.000307566 | 0.000236785 |
| k__Bacteria;p__Proteobacteria;c__Betaproteobacteria;o__Neisseriales;f__Neisseriaceae;g__Eikenella | 4.389162562 | 0.036168074 | 0.000473817 | 0.000231576 |
| k__Bacteria;p__Proteobacteria;c__Gammaproteobacteria;o__Cardiobacteriales;f__Cardiobacteriaceae;g__Cardiobacterium | 10.48320645 | 0.001204645 | 0.001264114 | 0.000427185 |
| k__Bacteria;p__Firmicutes;c__Bacilli;o__Bacillales;f__Staphylococcaceae;g__Staphylococcus | 9.851939004 | 0.001696528 | 0.001752189 | 4.22E-05 |
| k__Bacteria;p__Actinobacteria;c__Actinobacteria;o__Corynebacteriales;f__Corynebacteriaceae;g__Corynebacterium | 15.88421157 | 6.73E-05 | 0.003542379 | 0.001453431 |
| k__Bacteria;p__Fusobacteria;c__Fusobacteriia;o__Fusobacteriales;f__Leptotrichiaceae;g__Leptotrichia | 5.451609693 | 0.019550305 | 0.008958645 | 0.006480118 |
| k__Bacteria;p__Bacteroidetes;c__Flavobacteriia;o__Flavobacteriales;f__Flavobacteriaceae;g__Capnocytophaga | 9.589540727 | 0.001956889 | 0.007559186 | 0.004612355 |
| k__Bacteria;p__Proteobacteria;c__Betaproteobacteria;o__Burkholderiales;f__Burkholderiaceae;g__Lautropia | 12.09379509 | 0.000505899 | 0.006915191 | 0.001910146 |
| k__Bacteria;p__Proteobacteria;c__Gammaproteobacteria;o__Pasteurellales;f__Pasteurellaceae;g__Haemophilus | 10.5747624 | 0.00114642 | 0.150517209 | 0.096321197 |
| k__Bacteria;p__Firmicutes;c__Clostridia;o__Clostridiales;f__Lachnospiraceae_XIV;g__Lachnospiraceae_G-8 | 3.588778192 | 0.058170997 | 0.000341683 | 0.000196545 |
| k__Bacteria;p__Firmicutes;c__Clostridia;o__Clostridiales;f__Peptostreptococcaceae_XI;g__Filifactor | 3.441844149 | 0.063564705 | 0.001765344 | 0.001299868 |
| k__Bacteria;p__Firmicutes;c__Clostridia;o__Clostridiales;f__Peptoniphilaceae;g__Parvimonas | 3.035726725 | 0.08145007 | 0.000670291 | 0.000753269 |
| k__Bacteria;p__Proteobacteria;c__Betaproteobacteria;o__Burkholderiales;f__Comamonadaceae;g__Ottowia | 2.898793006 | 0.088645906 | 0.000214139 | 0.000161994 |
| k__Bacteria;p__Gracilibacteria_GN02;c__GN02_C-2;o__GN02_O-2;f__GN02_F-2;g__GN02_G-2 | 2.806556057 | 0.093879719 | 0.001390592 | 0.000431518 |
| k__Bacteria;p__Firmicutes;c__Clostridia;o__Clostridiales;f__Peptostreptococcaceae_XI;g__Peptostreptococcaceae_XIG-6 | 2.77334367 | 0.095845759 | 0.000448294 | 0.000481409 |
| k__Bacteria;p__Bacteroidetes;c__Flavobacteriia;o__Flavobacteriales;f__Flavobacteriaceae;g__Bergeyella | 2.655172414 | 0.103213565 | 0.005189718 | 0.003405158 |
| k__Bacteria;p__Proteobacteria;c__Gammaproteobacteria;o__Pseudomonadales;f__Moraxellaceae;g__Moraxella | 2.638891221 | 0.104276297 | 0.000655135 | 0.002963331 |
| k__Bacteria;p__Firmicutes;c__Bacilli;o__Bacillales;f__Gemellaceae;g__Gemella | 2.091108126 | 0.14815857 | 0.037997372 | 0.030801959 |
| k__Bacteria;p__Bacteroidetes;c__Bacteroidia;o__Bacteroidales;f__Porphyromonadaceae;g__Porphyromonas | 1.630143803 | 0.20168316 | 0.059764951 | 0.067842404 |
| k__Bacteria;p__Bacteroidetes;c__Bacteroidetes_C-1;o__Bacteroidetes_O-1;f__Bacteroidetes_F-1;g__Bacteroidetes_G-3 | 1.617383444 | 0.203457015 | 0.000264549 | 1.76E-05 |
| k__Bacteria;p__Bacteroidetes;c__Bacteroidetes_C-1;o__Bacteroidetes_O-1;f__Bacteroidetes_F-1;g__Bacteroidetes_G-5 | 1.591384689 | 0.207128412 | 0.000448494 | 8.03E-05 |
| k__Bacteria;p__Firmicutes;c__Negativicutes;o__Selenomonadales;f__Veillonellaceae;g__Megasphaera | 1.48926475 | 0.222330573 | 0.000767071 | 0.001570613 |
| k__Bacteria;p__SR1;c__SR1_C-1;o__SR1_O-1;f__SR1_F-1;g__SR1_G-1 | 1.438061789 | 0.230453243 | 0.003233288 | 0.00781292 |
| k__Bacteria;p__Firmicutes;c__Clostridia;o__Clostridiales;f__Ruminococcaceae;g__Ruminococcaceae_G-2 | 1.104692243 | 0.293238654 | 0.000727665 | 0.000955007 |
| k__Bacteria;p__Firmicutes;c__Clostridia;o__Clostridiales;f__Lachnospiraceae_XIV;g__Oribacterium | 1.07523511 | 0.299765945 | 0.007025805 | 0.006772234 |
| k__Bacteria;p__Firmicutes;c__Clostridia;o__Clostridiales;f__Lachnospiraceae_XIV;g__Lachnospiraceae_G-3 | 1.003586553 | 0.31644422 | 0.000435861 | 0.000355481 |
| k__Bacteria;p__Actinobacteria;c__Actinobacteria;o__Actinomycetales;f__Actinomycetaceae;g__Mobiluncus | 0.962902152 | 0.326456764 | 0.000211336 | 5.30E-05 |
| k__Bacteria;p__Proteobacteria;c__Gammaproteobacteria;o__Pasteurellales;f__Pasteurellaceae;g__Aggregatibacter | 0.906851769 | 0.340951119 | 0.010825674 | 0.008374268 |
| k__Bacteria;p__Synergistetes;c__Synergistia;o__Synergistales;f__Synergistaceae;g__Fretibacterium | 0.886566202 | 0.346409534 | 0.000180258 | 0.000153232 |
| k__Bacteria;p__Spirochaetes;c__Spirochaetia;o__Spirochaetales;f__Spirochaetaceae;g__Treponema | 0.854338822 | 0.355327797 | 0.001416435 | 0.000897701 |
| k__Bacteria;p__Fusobacteria;c__Fusobacteriia;o__Fusobacteriales;f__Fusobacteriaceae;g__Fusobacterium | 0.853908544 | 0.355448976 | 0.024466628 | 0.017861142 |
| k__Bacteria;p__Saccharibacteria_TM7;c__TM7_C-1;o__TM7_O-1;f__TM7_F-1;g__TM7_G-6 | 0.828242602 | 0.362781192 | 0.001819965 | 0.000979108 |
| k__Bacteria;p__Firmicutes;c__Negativicutes;o__Selenomonadales;f__Veillonellaceae;g__Selenomonas | 0.802557596 | 0.370329789 | 0.000731783 | 0.000663922 |
| k__Bacteria;p__Gracilibacteria_GN02;c__GN02_C-1;o__GN02_O-1;f__GN02_F-1;g__GN02_G-1 | 0.775048563 | 0.378659756 | 0.000205497 | 0.000167368 |
| k__Bacteria;p__Firmicutes;c__Clostridia;o__Clostridiales;f__Peptostreptococcaceae_XI;g__Peptostreptococcaceae_XIG-5 | 0.765108584 | 0.381734518 | 0.001054922 | 0.000748232 |
| k__Bacteria;p__Firmicutes;c__Clostridia;o__Clostridiales;f__Peptostreptococcaceae_XI;g__Peptostreptococcaceae_XIG-9 | 0.65863892 | 0.417040867 | 0.00015781 | 0.000118384 |
| k__Bacteria;p__Firmicutes;c__Clostridia;o__Clostridiales;f__Lachnospiraceae_XIV;g__Shuttleworthia | 0.547566148 | 0.459313873 | 0.000348708 | 0.000123688 |
| k__Bacteria;p__Bacteroidetes;c__Bacteroidia;o__Bacteroidales;f__Bacteroidales_F-2;g__Bacteroidales_G-2 | 0.468415242 | 0.493717136 | 0.000265185 | 0.000465931 |
| k__Bacteria;p__Actinobacteria;c__Coriobacteriia;o__Coriobacteriales;f__Coriobacteriaceae;g__Atopobium | 0.41205155 | 0.520930052 | 0.004381885 | 0.003134205 |
| k__Bacteria;p__Proteobacteria;c__Betaproteobacteria;o__Neisseriales;f__Neisseriaceae;g__Neisseria | 0.394138429 | 0.53013205 | 0.174889605 | 0.200064318 |
| k__Bacteria;p__Saccharibacteria_TM7;c__TM7_C-1;o__TM7_O-1;f__TM7_F-1;g__TM7_G-1 | 0.394138429 | 0.53013205 | 0.003887237 | 0.003905024 |
| k__Bacteria;p__Firmicutes;c__Clostridia;o__Clostridiales;f__Lachnospiraceae_XIV;g__Butyrivibrio | 0.359823531 | 0.548604258 | 0.000647767 | 0.000420992 |
| k__Bacteria;p__Firmicutes;c__Negativicutes;o__Selenomonadales;f__Veillonellaceae;g__Veillonella | 0.326466637 | 0.567747105 | 0.035641262 | 0.036475329 |
| k__Bacteria;p__Proteobacteria;c__Betaproteobacteria;o__Neisseriales;f__Neisseriaceae;g__Kingella | 0.310543862 | 0.577346641 | 0.001110564 | 0.0020688 |
| k__Bacteria;p__Firmicutes;c__Clostridia;o__Clostridiales;f__Ruminococcaceae;g__Ruminococcaceae_G-1 | 0.279892521 | 0.596771671 | 0.000635919 | 0.000819624 |
| k__Bacteria;p__Firmicutes;c__Bacilli;o__Lactobacillales;f__Streptococcaceae;g__Streptococcus | 0.150519978 | 0.698038943 | 0.266879269 | 0.254003622 |
| k__Bacteria;p__Fusobacteria;c__Fusobacteriia;o__Fusobacteriales;f__Leptotrichiaceae;g__Sneathia | 0.039508357 | 0.842444939 | 0.00029285 | 3.56E-06 |
| k__Bacteria;p__Actinobacteria;c__Actinobacteria;o__Actinomycetales;f__Micrococcaceae;g__Rothia | 0.026322337 | 0.871115602 | 0.028616374 | 0.029106959 |
| k__Bacteria;p__Proteobacteria;c__Gammaproteobacteria;o__Enterobacteriales;f__Enterobacteriaceae;g__Yersinia | 0.024134247 | 0.876543815 | 0.000267189 | 0.000309223 |
| k__Bacteria;p__Firmicutes;c__Clostridia;o__Clostridiales;f__Lachnospiraceae_XIV;g__Stomatobaculum | 0.021943574 | 0.882237306 | 0.00241326 | 0.001473705 |
| k__Bacteria;p__Firmicutes;c__Clostridia;o__Clostridiales;f__Lachnospiraceae_XIV;g__Lachnospiraceae_G-2 | 0.01630867 | 0.898382113 | 0.000300905 | 0.000127731 |
| k__Bacteria;p__Proteobacteria;c__Epsilonproteobacteria;o__Campylobacterales;f__Campylobacteraceae;g__Campylobacter | 0.014380256 | 0.90454834 | 0.003241035 | 0.003359432 |
| k__Bacteria;p__Firmicutes;c__Bacilli;o__Lactobacillales;f__Aerococcaceae;g__Abiotrophia | 0.006029302 | 0.9381076 | 0.00266469 | 0.002116182 |
| k__Bacteria;p__Bacteroidetes;c__Bacteroidia;o__Bacteroidales;f__Porphyromonadaceae;g__Tannerella | 0.004030452 | 0.949379632 | 0.000851375 | 0.000785285 |
| k__Bacteria;p__Firmicutes;c__Negativicutes;o__Selenomonadales;f__Veillonellaceae;g__Dialister | 0.004030452 | 0.949379632 | 0.001005098 | 0.001015184 |
| k__Bacteria;p__Actinobacteria;c__Actinobacteria;o__Actinomycetales;f__Actinomycetaceae;g__Actinomyces | 0.002438175 | 0.960618157 | 0.021728883 | 0.016586338 |
| k__Bacteria;p__Firmicutes;c__Clostridia;o__Clostridiales;f__Lachnospiraceae_XIV;Other | 0.001655967 | 0.96754018 | 0.000199418 | 3.58E-05 |
