# Supplementary material for: Characterization of the salivary microbiome in people with obesity
Source: PeerJ. 2018 Mar 16;6:e4458. doi: 10.7717/peerj.4458 (PMC5858547; doi:10.7717/peerj.4458)
Supplement: Table S7 [file peerj-06-4458-s009.docx]

| OTU | Test-Statistic | P | normal_weight_mean | obesity_mean |
| --- | --- | --- | --- | --- |
| k__Bacteria;p__Firmicutes;c__Bacilli;o__Bacillales;f__Gemellaceae;g__Gemella;s__Gemella_sanguinis | 29.35012501 | 6.04E-08 | 0.000414615 | 0 |
| k__Bacteria;p__SR1;c__SR1_C-1;o__SR1_O-1;f__SR1_F-1;g__SR1_G-1;s__SR1_G-1_sp._oral_taxon_874 | 27.05604431 | 1.98E-07 | 0.000422969 | 5.15E-06 |
| k__Bacteria;p__Bacteroidetes;c__Flavobacteriia;o__Flavobacteriales;f__Flavobacteriaceae;g__Capnocytophaga;s__Capnocytophaga_leadbetteri | 21.08777429 | 4.39E-06 | 0.002549288 | 0.000727574 |
| k__Bacteria;p__SR1;c__SR1_C-1;o__SR1_O-1;f__SR1_F-1;g__SR1_G-1;Other | 19.46659561 | 1.02E-05 | 0.000866561 | 8.27E-05 |
| k__Bacteria;p__Fusobacteria;c__Fusobacteriia;o__Fusobacteriales;f__Leptotrichiaceae;g__Leptotrichia;s__Leptotrichia_sp._oral_taxon_392 | 19.37971225 | 1.07E-05 | 0.000887667 | 0.000169425 |
| k__Bacteria;p__Bacteroidetes;c__Flavobacteriia;o__Flavobacteriales;f__Flavobacteriaceae;g__Capnocytophaga;s__Capnocytophaga_sp._oral_taxon_412 | 18.4806066 | 1.72E-05 | 0.000461271 | 8.66E-05 |
| k__Bacteria;p__Fusobacteria;c__Fusobacteriia;o__Fusobacteriales;f__Fusobacteriaceae;g__Fusobacterium;s__Fusobacterium_nucleatum_subsp._vincentii | 18.33353237 | 1.85E-05 | 0.001856621 | 0.004039185 |
| k__Bacteria;p__Actinobacteria;c__Actinobacteria;o__Actinomycetales;f__Micrococcaceae;g__Rothia;Other | 16.1099169 | 5.98E-05 | 0.005055856 | 0.001407432 |
| k__Bacteria;p__Proteobacteria;c__Gammaproteobacteria;o__Pasteurellales;f__Pasteurellaceae;g__Haemophilus;s__Haemophilus_parainfluenzae | 16.1099169 | 5.98E-05 | 0.129984576 | 0.070706185 |
| k__Bacteria;p__Bacteroidetes;c__Bacteroidia;o__Bacteroidales;f__Prevotellaceae;g__Alloprevotella;s__Alloprevotella_rava | 15.560485 | 7.99E-05 | 0.000133634 | 0.000745691 |
| k__Bacteria;p__Firmicutes;c__Erysipelotrichia;o__Erysipelotrichales;f__Erysipelotrichaceae;g__Solobacterium;s__Solobacterium_moorei | 15.5486391 | 8.04E-05 | 0.000755201 | 0.001506545 |
| k__Bacteria;p__Actinobacteria;c__Actinobacteria;o__Corynebacteriales;f__Corynebacteriaceae;g__Corynebacterium;s__Corynebacterium_durum | 15.32691446 | 9.04E-05 | 0.002621374 | 0.000727058 |
| k__Bacteria;p__Fusobacteria;c__Fusobacteriia;o__Fusobacteriales;f__Leptotrichiaceae;g__Leptotrichia;s__Leptotrichia_sp._oral_taxon_225 | 15.22492579 | 9.54E-05 | 0.001527866 | 0.000267241 |
| k__Bacteria;p__Fusobacteria;c__Fusobacteriia;o__Fusobacteriales;f__Fusobacteriaceae;g__Fusobacterium;Other | 13.0962587 | 0.000295886 | 0.007118946 | 0.002966229 |
| k__Bacteria;p__Proteobacteria;c__Gammaproteobacteria;o__Cardiobacteriales;f__Cardiobacteriaceae;g__Cardiobacterium;s__Cardiobacterium_hominis | 12.3899587 | 0.000431649 | 0.001026251 | 0.000287762 |
| k__Bacteria;p__Proteobacteria;c__Betaproteobacteria;o__Burkholderiales;f__Burkholderiaceae;g__Lautropia;s__Lautropia_mirabilis | 12.09379509 | 0.000505899 | 0.006915191 | 0.001910146 |
| k__Bacteria;p__Firmicutes;c__Clostridia;o__Clostridiales;f__Peptostreptococcaceae_XI;g__Mogibacterium;s__Mogibacterium_diversum | 11.03851321 | 0.000892384 | 0.000972043 | 0.001665186 |
| k__Bacteria;p__Firmicutes;c__Bacilli;o__Bacillales;f__Gemellaceae;g__Gemella;s__Gemella_morbillorum | 9.942480555 | 0.001615078 | 0.002846762 | 0.000795951 |
| k__Bacteria;p__Firmicutes;c__Bacilli;o__Bacillales;f__Staphylococcaceae;g__Staphylococcus;s__Staphylococcus_aureus | 9.851939004 | 0.001696528 | 0.001752189 | 4.22E-05 |
| k__Bacteria;p__Bacteroidetes;c__Flavobacteriia;o__Flavobacteriales;f__Flavobacteriaceae;g__Bergeyella;s__Bergeyella_sp._oral_taxon_322 | 9.243220381 | 0.002363689 | 0.002342527 | 0.001198847 |
| k__Bacteria;p__Firmicutes;c__Clostridia;o__Clostridiales;f__Peptostreptococcaceae_XI;g__Peptostreptococcus;s__Peptostreptococcus_stomatis | 9.243220381 | 0.002363689 | 0.001251882 | 0.002522018 |
| k__Bacteria;p__Fusobacteria;c__Fusobacteriia;o__Fusobacteriales;f__Leptotrichiaceae;Other;Other | 9.047599022 | 0.0026304 | 3.15E-05 | 0.000359819 |
| k__Bacteria;p__Firmicutes;c__Clostridia;o__Clostridiales;f__Peptostreptococcaceae_XI;g__Peptostreptococcaceae_XIG-1;s__Peptostreptococcaceae_XIG-1_Eubacterium_sulci | 8.98765985 | 0.002718089 | 0.002164788 | 0.004065674 |
| k__Bacteria;p__Firmicutes;c__Bacilli;o__Lactobacillales;f__Streptococcaceae;g__Streptococcus;Other | 8.569687018 | 0.003418059 | 0.029010229 | 0.016446529 |
| k__Bacteria;p__Firmicutes;c__Clostridia;o__Clostridiales;f__Lachnospiraceae_XIV;g__Catonella;s__Catonella_morbi | 8.405284371 | 0.003741319 | 0.001021683 | 0.001564876 |
| k__Bacteria;p__Proteobacteria;c__Betaproteobacteria;o__Neisseriales;f__Neisseriaceae;g__Neisseria;s__Neisseria_mucosa | 7.842414291 | 0.00510344 | 0.014260833 | 0.003100194 |
| k__Bacteria;p__Bacteroidetes;c__Bacteroidia;o__Bacteroidales;f__Prevotellaceae;g__Prevotella;s__Prevotella_scopos | 7.612217267 | 0.005797415 | 9.69E-06 | 0.000521852 |
| k__Bacteria;p__Proteobacteria;c__Epsilonproteobacteria;o__Campylobacterales;f__Campylobacteraceae;g__Campylobacter;s__Campylobacter_sp._oral_taxon_044 | 7.521247771 | 0.006097543 | 9.44E-05 | 0.000340902 |
| k__Bacteria;p__Bacteroidetes;c__Bacteroidia;o__Bacteroidales;f__Prevotellaceae;g__Prevotella;s__Prevotella_nanceiensis | 7.452306314 | 0.006335522 | 0.00781037 | 0.017474279 |
| k__Bacteria;p__Firmicutes;c__Clostridia;o__Clostridiales;f__Lachnospiraceae_XIV;g__Oribacterium;s__Oribacterium_asaccharolyticum | 7.2997849 | 0.006896287 | 0.001625281 | 0.000450563 |
| k__Bacteria;p__Proteobacteria;c__Gammaproteobacteria;o__Xanthomonadales;f__Xanthomonadaceae;g__Stenotrophomonas;s__Stenotrophomonas_maltophilia | 6.775190327 | 0.009243363 | 0.000307566 | 0.000236785 |
| k__Bacteria;p__Actinobacteria;c__Actinobacteria;o__Actinomycetales;f__Actinomycetaceae;g__Actinomyces;s__Actinomyces_lingnae_NVP | 6.722259107 | 0.009521694 | 0.00031143 | 0.001038652 |
| k__Bacteria;p__Fusobacteria;c__Fusobacteriia;o__Fusobacteriales;f__Fusobacteriaceae;g__Fusobacterium;s__Fusobacterium_sp._oral_taxon_203 | 6.659870814 | 0.009860806 | 0.00012623 | 0.000258922 |
| k__Bacteria;p__Firmicutes;c__Clostridia;o__Clostridiales;f__Peptostreptococcaceae_XI;g__Peptostreptococcaceae_XIG-7;Other | 6.629766672 | 0.01002884 | 0.000386009 | 0.000810139 |
| k__Bacteria;p__Bacteroidetes;c__Bacteroidia;o__Bacteroidales;f__Prevotellaceae;g__Prevotella;s__Prevotella_sp._oral_taxon_443 | 6.593176621 | 0.010237035 | 0.000222079 | 0.000740656 |
| k__Bacteria;p__Bacteroidetes;c__Bacteroidia;o__Bacteroidales;f__Prevotellaceae;g__Prevotella;s__Prevotella_pallens | 6.448886086 | 0.011102193 | 0.001838812 | 0.005141219 |
| k__Bacteria;p__Firmicutes;c__Bacilli;o__Lactobacillales;f__Carnobacteriaceae;g__Granulicatella;s__Granulicatella_adiacens | 6.34169279 | 0.011793228 | 0.013556993 | 0.025653483 |
| k__Bacteria;p__Bacteroidetes;c__Bacteroidia;o__Bacteroidales;f__Prevotellaceae;g__Alloprevotella;s__Alloprevotella_sp._oral_taxon_473 | 6.306373665 | 0.012030466 | 0.00267774 | 0.005268891 |
| k__Bacteria;p__Proteobacteria;c__Gammaproteobacteria;o__Pasteurellales;f__Pasteurellaceae;g__Aggregatibacter;Other | 6.200378166 | 0.012772302 | 0.0069781 | 0.003120107 |
| k__Bacteria;p__Firmicutes;c__Clostridia;o__Clostridiales;f__Lachnospiraceae_XIV;g__Lachnoanaerobaculum;s__Lachnoanaerobaculum_orale | 6.061266357 | 0.013817857 | 0.000151543 | 0.000278354 |
| k__Bacteria;p__Fusobacteria;c__Fusobacteriia;o__Fusobacteriales;f__Leptotrichiaceae;g__Leptotrichia;s__Leptotrichia_hongkongensis | 5.92252575 | 0.014948514 | 0.001016172 | 0.000422799 |
| k__Bacteria;p__Firmicutes;c__Clostridia;o__Clostridiales;f__Peptococcaceae;g__Peptococcus;Other | 5.85420524 | 0.015539874 | 0.000183946 | 0.000350759 |
| k__Bacteria;p__Proteobacteria;c__Gammaproteobacteria;o__Pasteurellales;f__Pasteurellaceae;g__Aggregatibacter;s__Aggregatibacter_aphrophilus | 5.844829927 | 0.015622884 | 0.000156585 | 0.000569454 |
| k__Bacteria;p__Bacteroidetes;c__Bacteroidia;o__Bacteroidales;f__Porphyromonadaceae;g__Porphyromonas;s__Porphyromonas_catoniae | 5.551018026 | 0.018469942 | 0.001228487 | 0.001828321 |
| k__Bacteria;p__Proteobacteria;c__Epsilonproteobacteria;o__Campylobacterales;f__Campylobacteraceae;g__Campylobacter;s__Campylobacter_concisus | 5.063492063 | 0.024434955 | 0.00135383 | 0.001775254 |
| k__Bacteria;p__Proteobacteria;c__Betaproteobacteria;o__Burkholderiales;f__Comamonadaceae;g__Delftia;s__Delftia_acidovorans | 5.000199035 | 0.025344404 | 0.000273061 | 0.000221118 |
| k__Bacteria;p__Proteobacteria;c__Gammaproteobacteria;o__Pasteurellales;f__Pasteurellaceae;g__Haemophilus;s__Haemophilus_sp._oral_taxon_036 | 4.813193187 | 0.028242665 | 0.006166204 | 0.003045713 |
| k__Bacteria;p__Bacteroidetes;c__Bacteroidia;o__Bacteroidales;f__Prevotellaceae;g__Prevotella;s__Prevotella_salivae | 4.68982303 | 0.030341772 | 0.001565771 | 0.002562958 |
| k__Bacteria;p__Firmicutes;c__Mollicutes;o__Mycoplasmatales;f__Mycoplasmataceae;g__Mycoplasma;s__Mycoplasma_faucium | 4.641428703 | 0.03120901 | 0.000126696 | 0.000143447 |
| k__Bacteria;p__Proteobacteria;c__Betaproteobacteria;o__Neisseriales;f__Neisseriaceae;Other;Other | 4.577977493 | 0.032385418 | 0.000436099 | 0.002079136 |
| k__Bacteria;p__Proteobacteria;c__Betaproteobacteria;o__Neisseriales;f__Neisseriaceae;g__Eikenella;s__Eikenella_corrodens | 4.389162562 | 0.036168074 | 0.000473817 | 0.000231576 |
| k__Bacteria;p__Proteobacteria;c__Betaproteobacteria;o__Neisseriales;f__Neisseriaceae;g__Neisseria;s__Neisseria_elongata | 4.271732099 | 0.03875141 | 0.009182604 | 0.002633158 |
| k__Bacteria;p__Bacteroidetes;c__Bacteroidia;o__Bacteroidales;f__Prevotellaceae;g__Prevotella;s__Prevotella_nigrescens | 4.214038443 | 0.040090783 | 0.000926314 | 0.000561133 |
| k__Bacteria;p__Proteobacteria;c__Gammaproteobacteria;o__Pseudomonadales;f__Moraxellaceae;g__Acinetobacter;s__Acinetobacter_baumannii | 4.155893915 | 0.041489868 | 0.000295862 | 0.00027077 |
| k__Bacteria;p__Proteobacteria;c__Betaproteobacteria;o__Neisseriales;f__Neisseriaceae;g__Neisseria;s__Neisseria_sp._oral_taxon_020 | 4.144625937 | 0.041766882 | 0.000402751 | 5.33E-05 |
| k__Bacteria;p__Actinobacteria;c__Actinobacteria;o__Actinomycetales;f__Actinomycetaceae;g__Actinomyces;s__Actinomyces_massiliensis | 4.021842659 | 0.044914616 | 0.000226202 | 9.58E-05 |
| k__Bacteria;p__Saccharibacteria_TM7;c__TM7_C-1;o__TM7_O-1;f__TM7_F-1;g__TM7_G-1;s__TM7_G-1_sp._oral_taxon_347 | 4.011422952 | 0.045192994 | 0.000264044 | 6.32E-05 |
| k__Bacteria;p__Saccharibacteria_TM7;c__TM7_C-1;o__TM7_O-1;f__TM7_F-1;g__TM7_G-3;s__TM7_G-3_sp._oral_taxon_351 | 3.873264666 | 0.049061003 | 0.000857292 | 0.001186628 |
| k__Bacteria;p__Fusobacteria;c__Fusobacteriia;o__Fusobacteriales;f__Leptotrichiaceae;g__Leptotrichia;s__Leptotrichia_sp._oral_taxon_218 | 3.793585372 | 0.051449331 | 0.00020227 | 0.0003645 |
| k__Bacteria;p__SR1;c__SR1_C-1;o__SR1_O-1;f__SR1_F-1;g__SR1_G-1;s__SR1_G-1_sp._oral_taxon_875 | 3.768313485 | 0.052232273 | 0.001063958 | 0.006390812 |
| k__Bacteria;p__Fusobacteria;c__Fusobacteriia;o__Fusobacteriales;f__Leptotrichiaceae;g__Leptotrichia;s__Leptotrichia_sp._oral_taxon_215 | 3.627498423 | 0.05683218 | 0.000431115 | 0.000877061 |
| k__Bacteria;p__Firmicutes;c__Clostridia;o__Clostridiales;f__Lachnospiraceae_XIV;g__Lachnospiraceae_G-8;s__Lachnospiraceae_G-8_sp._oral_taxon_500 | 3.588778192 | 0.058170997 | 0.000341683 | 0.000196545 |
| k__Bacteria;p__Bacteroidetes;c__Bacteroidia;o__Bacteroidales;f__Prevotellaceae;g__Prevotella;s__Prevotella_intermedia | 3.58006151 | 0.058476986 | 0.000374992 | 0.000634531 |
| k__Bacteria;p__Bacteroidetes;c__Bacteroidia;o__Bacteroidales;f__Prevotellaceae;g__Prevotella;s__Prevotella_melaninogenica | 3.494302632 | 0.061580339 | 0.043564963 | 0.059879393 |
| k__Bacteria;p__Firmicutes;c__Clostridia;o__Clostridiales;f__Peptostreptococcaceae_XI;g__Filifactor;s__Filifactor_alocis | 3.441844149 | 0.063564705 | 0.001765344 | 0.001299868 |
| k__Bacteria;p__Firmicutes;c__Negativicutes;o__Selenomonadales;f__Veillonellaceae;g__Veillonella;s__Veillonella_dispar | 3.337861372 | 0.067702552 | 0.011867305 | 0.018074875 |
| k__Bacteria;p__Bacteroidetes;c__Bacteroidia;o__Bacteroidales;f__Prevotellaceae;g__Prevotella;s__Prevotella_sp._oral_taxon_472 | 3.235639026 | 0.072052189 | 0.000554594 | 0.000243022 |
| k__Bacteria;p__Actinobacteria;c__Actinobacteria;o__Corynebacteriales;f__Corynebacteriaceae;g__Corynebacterium;s__Corynebacterium_matruchotii | 3.235557546 | 0.072055773 | 0.000902902 | 0.000415335 |
| k__Bacteria;p__Bacteroidetes;c__Bacteroidia;o__Bacteroidales;f__Prevotellaceae;g__Prevotella;s__Prevotella_pleuritidis | 3.224253599 | 0.072554866 | 8.65E-05 | 0.000235527 |
| k__Bacteria;p__Firmicutes;c__Negativicutes;o__Selenomonadales;f__Veillonellaceae;g__Veillonella;s__Veillonella_parvula | 3.162662077 | 0.075340235 | 0.002266175 | 0.002447907 |
| k__Bacteria;p__Firmicutes;c__Negativicutes;o__Selenomonadales;f__Veillonellaceae;g__Veillonella;s__Veillonella_sp._oral_taxon_780 | 3.063810303 | 0.080053742 | 0.003322233 | 0.002019562 |
| k__Bacteria;p__Spirochaetes;c__Spirochaetia;o__Spirochaetales;f__Spirochaetaceae;g__Treponema;Other | 3.040015881 | 0.081235122 | 0.000183408 | 0.000112456 |
| k__Bacteria;p__Firmicutes;c__Clostridia;o__Clostridiales;f__Peptoniphilaceae;g__Parvimonas;s__Parvimonas_micra | 3.035726725 | 0.08145007 | 0.000670291 | 0.000753269 |
| k__Bacteria;p__Firmicutes;c__Clostridia;o__Clostridiales;f__Lachnospiraceae_XIV;g__Oribacterium;Other | 2.986764194 | 0.083947761 | 0.005400525 | 0.006321671 |
| k__Bacteria;p__Proteobacteria;c__Betaproteobacteria;o__Burkholderiales;f__Comamonadaceae;g__Ottowia;s__Ottowia_sp._oral_taxon_894 | 2.898793006 | 0.088645906 | 0.000214139 | 0.000161994 |
| k__Bacteria;p__Firmicutes;c__Negativicutes;o__Selenomonadales;f__Veillonellaceae;g__Veillonella;Other | 2.890033338 | 0.089129081 | 0.012608987 | 0.006513019 |
| k__Bacteria;p__Gracilibacteria_GN02;c__GN02_C-2;o__GN02_O-2;f__GN02_F-2;g__GN02_G-2;s__GN02_G-2_sp._oral_taxon_873 | 2.806556057 | 0.093879719 | 0.001390592 | 0.000431518 |
| k__Bacteria;p__Firmicutes;c__Negativicutes;o__Selenomonadales;f__Veillonellaceae;g__Selenomonas;s__Selenomonas_noxia | 2.796303087 | 0.094481929 | 0.000342878 | 0.000174867 |
| k__Bacteria;p__Firmicutes;c__Clostridia;o__Clostridiales;f__Peptostreptococcaceae_XI;g__Peptostreptococcaceae_XIG-6;s__Peptostreptococcaceae_XIG-6_Eubacterium_nodatum | 2.77334367 | 0.095845759 | 0.000448294 | 0.000481409 |
| k__Bacteria;p__Firmicutes;c__Bacilli;o__Lactobacillales;f__Streptococcaceae;g__Streptococcus;s__Streptococcus_parasanguinis_II | 2.70134846 | 0.100263413 | 0.008183643 | 0.011510535 |
| k__Bacteria;p__Bacteroidetes;c__Bacteroidia;o__Bacteroidales;f__Prevotellaceae;g__Prevotella;s__Prevotella_shahii | 2.655841206 | 0.103170165 | 0.000815596 | 0.001257159 |
| k__Bacteria;p__Proteobacteria;c__Gammaproteobacteria;o__Pseudomonadales;f__Moraxellaceae;g__Moraxella;s__Moraxella_catarrhalis | 2.638891221 | 0.104276297 | 0.000655135 | 0.002963331 |
| k__Bacteria;p__Bacteroidetes;c__Flavobacteriia;o__Flavobacteriales;f__Flavobacteriaceae;g__Capnocytophaga;s__Capnocytophaga_sputigena | 2.474448923 | 0.115709992 | 0.002166401 | 0.001399748 |
| k__Bacteria;p__Proteobacteria;c__Gammaproteobacteria;o__Pasteurellales;f__Pasteurellaceae;g__Haemophilus;s__Haemophilus_sputorum | 2.386535691 | 0.122384573 | 0.003877404 | 0.007389004 |
| k__Bacteria;p__Bacteroidetes;c__Bacteroidia;o__Bacteroidales;f__Prevotellaceae;g__Prevotella;s__Prevotella_veroralis | 2.344266696 | 0.125744721 | 0.000592806 | 0.003584835 |
| k__Bacteria;p__Bacteroidetes;c__Bacteroidia;o__Bacteroidales;f__Prevotellaceae;g__Alloprevotella;s__Alloprevotella_tannerae | 2.236410002 | 0.134793613 | 0.001112055 | 0.002634106 |
| k__Bacteria;p__Bacteroidetes;c__Bacteroidia;o__Bacteroidales;f__Prevotellaceae;g__Prevotella;s__Prevotella_aurantiaca | 2.224548812 | 0.135832333 | 0.00182046 | 0.00371386 |
| k__Bacteria;p__Proteobacteria;c__Epsilonproteobacteria;o__Campylobacterales;f__Campylobacteraceae;g__Campylobacter;s__Campylobacter_rectus | 2.050505051 | 0.152155708 | 0.001792849 | 0.001243276 |
| k__Bacteria;p__Bacteroidetes;c__Bacteroidia;o__Bacteroidales;f__Prevotellaceae;g__Prevotella;s__Prevotella_denticola | 2.01313895 | 0.155942379 | 0.000260346 | 0.000508693 |
| k__Bacteria;p__Proteobacteria;c__Gammaproteobacteria;o__Cardiobacteriales;f__Cardiobacteriaceae;g__Cardiobacterium;s__Cardiobacterium_valvarum | 1.931278775 | 0.164618608 | 0.000237862 | 0.000139422 |
| k__Bacteria;p__Firmicutes;c__Clostridia;o__Clostridiales;f__Lachnospiraceae_XIV;g__Lachnoanaerobaculum;s__Lachnoanaerobaculum_umeaense | 1.853460716 | 0.173381429 | 0.00117574 | 0.001653499 |
| k__Bacteria;p__Proteobacteria;c__Betaproteobacteria;o__Neisseriales;f__Neisseriaceae;g__Kingella;s__Kingella_sp._oral_taxon_012 | 1.815428921 | 0.17785835 | 0.000793186 | 0.001819411 |
| k__Bacteria;p__Bacteroidetes;c__Bacteroidia;o__Bacteroidales;f__Porphyromonadaceae;g__Porphyromonas;s__Porphyromonas_sp._oral_taxon_930 | 1.776515612 | 0.182577772 | 0.001619722 | 0.000834817 |
| k__Bacteria;p__Firmicutes;c__Bacilli;o__Lactobacillales;f__Carnobacteriaceae;g__Granulicatella;s__Granulicatella_elegans | 1.740054765 | 0.187131888 | 0.003353853 | 0.004507209 |
| k__Bacteria;p__Proteobacteria;c__Gammaproteobacteria;o__Pasteurellales;f__Pasteurellaceae;g__Haemophilus;s__Haemophilus_paraphrohaemolyticus | 1.73266008 | 0.188071537 | 0.001305398 | 0.003095223 |
| k__Bacteria;p__Proteobacteria;c__Betaproteobacteria;o__Neisseriales;f__Neisseriaceae;g__Neisseria;Other | 1.630143803 | 0.20168316 | 0.011305743 | 0.006109251 |
| k__Bacteria;p__Bacteroidetes;c__Bacteroidetes_C-1;o__Bacteroidetes_O-1;f__Bacteroidetes_F-1;g__Bacteroidetes_G-3;s__Bacteroidetes_G-3_sp._oral_taxon_503 | 1.617383444 | 0.203457015 | 0.000264549 | 1.76E-05 |
| k__Bacteria;p__Bacteroidetes;c__Bacteroidetes_C-1;o__Bacteroidetes_O-1;f__Bacteroidetes_F-1;g__Bacteroidetes_G-5;s__Bacteroidetes_G-5_sp._oral_taxon_505 | 1.591384689 | 0.207128412 | 0.000448494 | 8.03E-05 |
| k__Bacteria;p__Bacteroidetes;c__Bacteroidia;o__Bacteroidales;f__Prevotellaceae;g__Prevotella;s__Prevotella_oris | 1.558889386 | 0.21182791 | 0.000801825 | 0.001642612 |
| k__Bacteria;p__Bacteroidetes;c__Flavobacteriia;o__Flavobacteriales;f__Flavobacteriaceae;g__Capnocytophaga;s__Capnocytophaga_granulosa | 1.558889386 | 0.21182791 | 0.001223017 | 0.001008504 |
| k__Bacteria;p__Firmicutes;c__Negativicutes;o__Selenomonadales;f__Veillonellaceae;g__Megasphaera;s__Megasphaera_micronuciformis | 1.48926475 | 0.222330573 | 0.000767071 | 0.001570613 |
| k__Bacteria;p__Bacteroidetes;c__Bacteroidia;o__Bacteroidales;f__Porphyromonadaceae;g__Porphyromonas;s__Porphyromonas_sp._oral_taxon_279 | 1.454993283 | 0.227728425 | 0.050072395 | 0.059049809 |
| k__Bacteria;p__Actinobacteria;c__Actinobacteria;o__Actinomycetales;f__Actinomycetaceae;g__Actinomyces;s__Actinomyces_sp._oral_taxon_180 | 1.421157387 | 0.233212997 | 0.013246598 | 0.008783366 |
| k__Bacteria;p__Bacteroidetes;c__Bacteroidia;o__Bacteroidales;f__Prevotellaceae;g__Alloprevotella;s__Alloprevotella_sp._oral_taxon_308 | 1.380157615 | 0.240074302 | 6.54E-05 | 0.000595492 |
| k__Bacteria;p__Actinobacteria;c__Actinobacteria;o__Actinomycetales;f__Micrococcaceae;g__Rothia;s__Rothia_aeria | 1.354679803 | 0.244461651 | 0.00408183 | 0.006316646 |
| k__Bacteria;p__Actinobacteria;c__Actinobacteria;o__Corynebacteriales;f__Corynebacteriaceae;g__Corynebacterium;s__Corynebacterium_diphtheriae | 1.337736036 | 0.247433618 | 1.81E-05 | 0.000311039 |
| k__Bacteria;p__Saccharibacteria_TM7;c__TM7_C-1;o__TM7_O-1;f__TM7_F-1;g__TM7_G-1;s__TM7_G-1_sp._oral_taxon_346 | 1.226625023 | 0.268064396 | 0.000264051 | 0.000236172 |
| k__Bacteria;p__Firmicutes;c__Negativicutes;o__Selenomonadales;f__Veillonellaceae;g__Veillonella;s__Veillonella_atypica | 1.195482162 | 0.274226522 | 0.002948362 | 0.005350135 |
| k__Bacteria;p__Fusobacteria;c__Fusobacteriia;o__Fusobacteriales;f__Leptotrichiaceae;g__Leptotrichia;s__Leptotrichia_sp._oral_taxon_221 | 1.195482162 | 0.274226522 | 0.000630122 | 0.000613041 |
| k__Bacteria;p__Fusobacteria;c__Fusobacteriia;o__Fusobacteriales;f__Leptotrichiaceae;g__Leptotrichia;Other | 1.195452058 | 0.274232564 | 0.003954236 | 0.003272184 |
| k__Bacteria;p__Bacteroidetes;c__Bacteroidia;o__Bacteroidales;f__Prevotellaceae;g__Alloprevotella;s__Alloprevotella_sp._oral_taxon_912 | 1.147697513 | 0.28403166 | 0.000155987 | 0.000159169 |
| k__Bacteria;p__Proteobacteria;c__Betaproteobacteria;o__Neisseriales;f__Neisseriaceae;g__Kingella;s__Kingella_oralis | 1.104720062 | 0.293232577 | 0.000317378 | 0.00024939 |
| k__Bacteria;p__Firmicutes;c__Clostridia;o__Clostridiales;f__Ruminococcaceae;g__Ruminococcaceae_G-2;s__Ruminococcaceae_G-2_sp._oral_taxon_085 | 1.104692243 | 0.293238654 | 0.000727665 | 0.000955007 |
| k__Bacteria;p__Bacteroidetes;c__Bacteroidia;o__Bacteroidales;f__Prevotellaceae;g__Prevotella;s__Prevotella_sp._oral_taxon_309 | 1.049771787 | 0.305559652 | 0.000152643 | 0.000170408 |
| k__Bacteria;p__Firmicutes;c__Clostridia;o__Clostridiales;f__Peptostreptococcaceae_XI;g__Peptostreptococcaceae_XIG-5;s__Peptostreptococcaceae_XIG-5_sp._oral_taxon_493 | 1.047653435 | 0.306048141 | 0.000363903 | 0.00049949 |
| k__Bacteria;p__Firmicutes;c__Clostridia;o__Clostridiales;f__Lachnospiraceae_XIV;g__Lachnospiraceae_G-3;s__Lachnospiraceae_G-3_sp._oral_taxon_100 | 1.003586553 | 0.31644422 | 0.000435861 | 0.000355481 |
| k__Bacteria;p__Actinobacteria;c__Actinobacteria;o__Actinomycetales;f__Actinomycetaceae;g__Actinomyces;s__Actinomyces_sp._oral_taxon_169 | 0.989252127 | 0.31992523 | 0.002674048 | 0.004535116 |
| k__Bacteria;p__Actinobacteria;c__Actinobacteria;o__Actinomycetales;f__Actinomycetaceae;g__Mobiluncus;s__Mobiluncus_mulieris | 0.962902152 | 0.326456764 | 0.000211336 | 5.30E-05 |
| k__Bacteria;p__Firmicutes;c__Bacilli;o__Bacillales;f__Gemellaceae;g__Gemella;Other | 0.961387272 | 0.326837603 | 0.034735995 | 0.030006008 |
| k__Bacteria;p__Spirochaetes;c__Spirochaetia;o__Spirochaetales;f__Spirochaetaceae;g__Treponema;s__Treponema_sp._oral_taxon_257 | 0.952228382 | 0.32915273 | 0.000409934 | 0.000184662 |
| k__Bacteria;p__Firmicutes;c__Clostridia;o__Clostridiales;f__Peptostreptococcaceae_XI;g__Peptostreptococcaceae_XIG-5;s__Peptostreptococcaceae_XIG-5_Eubacterium_saphenum | 0.945095202 | 0.330970922 | 0.000691018 | 0.000248741 |
| k__Bacteria;p__Synergistetes;c__Synergistia;o__Synergistales;f__Synergistaceae;g__Fretibacterium;Other | 0.886566202 | 0.346409534 | 0.000180258 | 0.000153232 |
| k__Bacteria;p__Bacteroidetes;c__Bacteroidia;o__Bacteroidales;f__Prevotellaceae;g__Prevotella;s__Prevotella_micans | 0.868832901 | 0.351278614 | 0.000129863 | 0.000173865 |
| k__Bacteria;p__Bacteroidetes;c__Flavobacteriia;o__Flavobacteriales;f__Flavobacteriaceae;g__Capnocytophaga;s__Capnocytophaga_sp._oral_taxon_332 | 0.842460085 | 0.358694132 | 0.000354992 | 8.45E-05 |
| k__Bacteria;p__Saccharibacteria_TM7;c__TM7_C-1;o__TM7_O-1;f__TM7_F-1;g__TM7_G-6;s__TM7_G-6_sp._oral_taxon_870 | 0.828242602 | 0.362781192 | 0.001819965 | 0.000979108 |
| k__Bacteria;p__Firmicutes;c__Clostridia;o__Clostridiales;f__Lachnospiraceae_XIV;g__Stomatobaculum;s__Stomatobaculum_longum | 0.793264683 | 0.373114779 | 0.000226549 | 0.000396315 |
| k__Bacteria;p__Bacteroidetes;c__Bacteroidia;o__Bacteroidales;f__Porphyromonadaceae;g__Porphyromonas;s__Porphyromonas_endodontalis | 0.777479226 | 0.377913193 | 0.003027013 | 0.003563719 |
| k__Bacteria;p__Gracilibacteria_GN02;c__GN02_C-1;o__GN02_O-1;f__GN02_F-1;g__GN02_G-1;s__GN02_G-1_sp._oral_taxon_872 | 0.775048563 | 0.378659756 | 0.000205497 | 0.000167368 |
| k__Bacteria;p__Proteobacteria;c__Betaproteobacteria;o__Neisseriales;f__Neisseriaceae;g__Neisseria;s__Neisseria_oralis | 0.752874761 | 0.385567588 | 0.001914997 | 0.001615046 |
| k__Bacteria;p__Firmicutes;c__Negativicutes;o__Selenomonadales;f__Veillonellaceae;g__Veillonella;s__Veillonella_rogosae | 0.704703515 | 0.401207744 | 0.002628201 | 0.002069831 |
| k__Bacteria;p__Proteobacteria;c__Gammaproteobacteria;o__Pasteurellales;f__Pasteurellaceae;g__Haemophilus;s__Haemophilus_parahaemolyticus | 0.684941031 | 0.407890472 | 0.000961608 | 0.004632917 |
| k__Bacteria;p__Proteobacteria;c__Betaproteobacteria;o__Neisseriales;f__Neisseriaceae;g__Neisseria;s__Neisseria_subflava | 0.68114644 | 0.409192231 | 0.078545007 | 0.110961977 |
| k__Bacteria;p__Firmicutes;c__Clostridia;o__Clostridiales;f__Peptostreptococcaceae_XI;g__Peptostreptococcaceae_XIG-9;s__Peptostreptococcaceae_XIG-9_Eubacterium_brachy | 0.65863892 | 0.417040867 | 0.00015781 | 0.000118384 |
| k__Bacteria;p__Proteobacteria;c__Gammaproteobacteria;o__Pasteurellales;f__Pasteurellaceae;g__Aggregatibacter;s__Aggregatibacter_sp._oral_taxon_512 | 0.613092017 | 0.433625981 | 0.003690989 | 0.004684707 |
| k__Bacteria;p__Firmicutes;c__Clostridia;o__Clostridiales;f__Lachnospiraceae_XIV;g__Shuttleworthia;s__Shuttleworthia_satelles | 0.547566148 | 0.459313873 | 0.000348708 | 0.000123688 |
| k__Bacteria;p__Firmicutes;c__Negativicutes;o__Selenomonadales;f__Veillonellaceae;g__Selenomonas;s__Selenomonas_sp._oral_taxon_136 | 0.52835541 | 0.467299101 | 0.000271009 | 0.000340111 |
| k__Bacteria;p__Firmicutes;c__Bacilli;o__Lactobacillales;f__Streptococcaceae;g__Streptococcus;s__Streptococcus_anginosus | 0.527889735 | 0.467495412 | 0.000524043 | 0.000764135 |
| k__Bacteria;p__Fusobacteria;c__Fusobacteriia;o__Fusobacteriales;f__Fusobacteriaceae;g__Fusobacterium;s__Fusobacterium_periodonticum | 0.527889735 | 0.467495412 | 0.01536483 | 0.010596806 |
| k__Bacteria;p__Bacteroidetes;c__Bacteroidia;o__Bacteroidales;f__Prevotellaceae;g__Prevotella;s__Prevotella_sp._oral_taxon_304 | 0.521496434 | 0.470204043 | 0.001341465 | 0.00037625 |
| k__Bacteria;p__SR1;c__SR1_C-1;o__SR1_O-1;f__SR1_F-1;g__SR1_G-1;s__SR1_G-1_sp._oral_taxon_345 | 0.507639331 | 0.476162561 | 0.0008798 | 0.001334283 |
| k__Bacteria;p__Bacteroidetes;c__Bacteroidia;o__Bacteroidales;f__Prevotellaceae;g__Prevotella;s__Prevotella_sp._oral_taxon_313 | 0.473975505 | 0.491163913 | 0.000464482 | 0.000285936 |
| k__Bacteria;p__Bacteroidetes;c__Bacteroidia;o__Bacteroidales;f__Bacteroidales_F-2;g__Bacteroidales_G-2;s__Bacteroidales_G-2_sp._oral_taxon_274 | 0.468415242 | 0.493717136 | 0.000265185 | 0.000465931 |
| k__Bacteria;p__Proteobacteria;c__Gammaproteobacteria;o__Pasteurellales;f__Pasteurellaceae;g__Haemophilus;s__Haemophilus_sp._oral_taxon_908 | 0.453226376 | 0.500806769 | 0.000776549 | 0.001808777 |
| k__Bacteria;p__Spirochaetes;c__Spirochaetia;o__Spirochaetales;f__Spirochaetaceae;g__Treponema;s__Treponema_sp._oral_taxon_238 | 0.414347044 | 0.519771323 | 0.000330388 | 0.000220437 |
| k__Bacteria;p__Actinobacteria;c__Coriobacteriia;o__Coriobacteriales;f__Coriobacteriaceae;g__Atopobium;s__Atopobium_parvulum | 0.41205155 | 0.520930052 | 0.004381885 | 0.003134205 |
| k__Bacteria;p__Firmicutes;c__Negativicutes;o__Selenomonadales;f__Veillonellaceae;g__Selenomonas;s__Selenomonas_sputigena | 0.395333059 | 0.529509356 | 0.000117897 | 0.000148945 |
| k__Bacteria;p__Firmicutes;c__Clostridia;o__Clostridiales;f__Lachnospiraceae_XIV;g__Stomatobaculum;s__Stomatobaculum_sp._oral_taxon_097 | 0.394148354 | 0.530126871 | 0.00218671 | 0.00107739 |
| k__Bacteria;p__Firmicutes;c__Clostridia;o__Clostridiales;f__Lachnospiraceae_XIV;g__Butyrivibrio;s__Butyrivibrio_sp._oral_taxon_455 | 0.359823531 | 0.548604258 | 0.000647767 | 0.000420992 |
| k__Bacteria;p__Fusobacteria;c__Fusobacteriia;o__Fusobacteriales;f__Leptotrichiaceae;g__Leptotrichia;s__Leptotrichia_sp._oral_taxon_219 | 0.340406322 | 0.559594779 | 0.00010542 | 0.000361146 |
| k__Bacteria;p__Actinobacteria;c__Actinobacteria;o__Actinomycetales;f__Actinomycetaceae;g__Actinomyces;s__Actinomyces_graevenitzii | 0.334585726 | 0.562971242 | 0.005270605 | 0.002133419 |
| k__Bacteria;p__Bacteroidetes;c__Bacteroidia;o__Bacteroidales;f__Prevotellaceae;g__Alloprevotella;s__Alloprevotella_sp._oral_taxon_914 | 0.295019157 | 0.587021944 | 0.006523323 | 0.009836667 |
| k__Bacteria;p__Firmicutes;c__Clostridia;o__Clostridiales;f__Peptostreptococcaceae_XI;g__Peptostreptococcaceae_XIG-1;s__Peptostreptococcaceae_XIG-1_Eubacterium_infirmum | 0.289005786 | 0.590858351 | 0.000247091 | 0.000106453 |
| k__Bacteria;p__Firmicutes;c__Clostridia;o__Clostridiales;f__Ruminococcaceae;g__Ruminococcaceae_G-1;s__Ruminococcaceae_G-1_sp._oral_taxon_075 | 0.279892521 | 0.596771671 | 0.000635919 | 0.000819624 |
| k__Bacteria;p__Spirochaetes;c__Spirochaetia;o__Spirochaetales;f__Spirochaetaceae;g__Treponema;s__Treponema_denticola | 0.274352565 | 0.600426866 | 0.000294199 | 0.00024349 |
| k__Bacteria;p__Bacteroidetes;c__Bacteroidia;o__Bacteroidales;f__Prevotellaceae;g__Prevotella;Other | 0.250896638 | 0.616444433 | 0.004061263 | 0.007237319 |
| k__Bacteria;p__Bacteroidetes;c__Flavobacteriia;o__Flavobacteriales;f__Flavobacteriaceae;g__Bergeyella;Other | 0.240236503 | 0.624035352 | 0.002516408 | 0.001824411 |
| k__Bacteria;p__Bacteroidetes;c__Flavobacteriia;o__Flavobacteriales;f__Flavobacteriaceae;g__Capnocytophaga;Other | 0.238619576 | 0.625204912 | 0.000804217 | 0.001305399 |
| k__Bacteria;p__Proteobacteria;c__Gammaproteobacteria;o__Pasteurellales;f__Pasteurellaceae;g__Haemophilus;Other | 0.236906996 | 0.626449025 | 0.005017548 | 0.004831568 |
| k__Bacteria;p__Firmicutes;c__Bacilli;o__Lactobacillales;f__Streptococcaceae;g__Streptococcus;s__Streptococcus_vestibularis | 0.23690103 | 0.626453369 | 0.030719401 | 0.020878062 |
| k__Bacteria;p__Bacteroidetes;c__Flavobacteriia;o__Flavobacteriales;f__Flavobacteriaceae;g__Bergeyella;s__Bergeyella_sp._oral_taxon_907 | 0.228953433 | 0.63230093 | 0.000330784 | 0.0003819 |
| k__Bacteria;p__Bacteroidetes;c__Bacteroidia;o__Bacteroidales;f__Prevotellaceae;g__Prevotella;s__Prevotella_baroniae | 0.217515404 | 0.640939612 | 0.000158166 | 0.00021817 |
| k__Bacteria;p__Bacteroidetes;c__Bacteroidia;o__Bacteroidales;f__Porphyromonadaceae;g__Tannerella;s__Tannerella_sp._oral_taxon_286 | 0.191277146 | 0.661855779 | 0.000412168 | 0.000476896 |
| k__Bacteria;p__Firmicutes;c__Negativicutes;o__Selenomonadales;f__Veillonellaceae;g__Dialister;s__Dialister_pneumosintes | 0.185544492 | 0.666651063 | 0.000314375 | 0.000207535 |
| k__Bacteria;p__Firmicutes;c__Negativicutes;o__Selenomonadales;f__Veillonellaceae;g__Dialister;s__Dialister_invisus | 0.173209932 | 0.677274655 | 0.000690723 | 0.000807649 |
| k__Bacteria;p__Bacteroidetes;c__Bacteroidia;o__Bacteroidales;f__Porphyromonadaceae;g__Porphyromonas;s__Porphyromonas_gingivalis | 0.1684508 | 0.681492316 | 0.001178215 | 0.001166352 |
| k__Bacteria;p__Bacteroidetes;c__Bacteroidia;o__Bacteroidales;f__Prevotellaceae;g__Prevotella;s__Prevotella_sp._oral_taxon_396 | 0.134550833 | 0.71375947 | 0.000568316 | 0.000617275 |
| k__Bacteria;p__Spirochaetes;c__Spirochaetia;o__Spirochaetales;f__Spirochaetaceae;g__Treponema;s__Treponema_sp._oral_taxon_262 | 0.132659363 | 0.7156905 | 0.000198506 | 0.000136656 |
| k__Bacteria;p__Firmicutes;c__Bacilli;o__Lactobacillales;f__Streptococcaceae;g__Streptococcus;s__Streptococcus_sp._oral_taxon_058 | 0.092003782 | 0.761645052 | 0.198214393 | 0.204265518 |
| k__Bacteria;p__Fusobacteria;c__Fusobacteriia;o__Fusobacteriales;f__Leptotrichiaceae;g__Sneathia;Other | 0.039508357 | 0.842444939 | 0.00029285 | 3.56E-06 |
| k__Bacteria;p__Firmicutes;c__Bacilli;o__Lactobacillales;f__Streptococcaceae;g__Streptococcus;s__Streptococcus_constellatus | 0.035537944 | 0.850472877 | 0.000227561 | 0.000138842 |
| k__Bacteria;p__Bacteroidetes;c__Bacteroidia;o__Bacteroidales;f__Porphyromonadaceae;g__Porphyromonas;s__Porphyromonas_sp._oral_taxon_278 | 0.031099169 | 0.860019325 | 0.002639119 | 0.001399385 |
| k__Bacteria;p__Proteobacteria;c__Gammaproteobacteria;o__Enterobacteriales;f__Enterobacteriaceae;g__Yersinia;s__Yersinia_pestis | 0.024134247 | 0.876543815 | 0.000267189 | 0.000309223 |
| k__Bacteria;p__Saccharibacteria_TM7;c__TM7_C-1;o__TM7_O-1;f__TM7_F-1;g__TM7_G-1;s__TM7_G-1_sp._oral_taxon_352 | 0.021943574 | 0.882237306 | 0.003359142 | 0.003605604 |
| k__Bacteria;p__Firmicutes;c__Clostridia;o__Clostridiales;f__Lachnospiraceae_XIV;g__Lachnospiraceae_G-2;s__Lachnospiraceae_G-2_sp._oral_taxon_096 | 0.01630867 | 0.898382113 | 0.000300905 | 0.000127731 |
| k__Bacteria;p__Bacteroidetes;c__Bacteroidia;o__Bacteroidales;f__Porphyromonadaceae;g__Tannerella;s__Tannerella_forsythia | 0.008411333 | 0.926925813 | 0.000439208 | 0.000308389 |
| k__Bacteria;p__Fusobacteria;c__Fusobacteriia;o__Fusobacteriales;f__Leptotrichiaceae;g__Leptotrichia;s__Leptotrichia_sp._oral_taxon_498 | 0.006064476 | 0.93792769 | 0.000203777 | 0.000132721 |
| k__Bacteria;p__Firmicutes;c__Bacilli;o__Lactobacillales;f__Aerococcaceae;g__Abiotrophia;s__Abiotrophia_defectiva | 0.006029302 | 0.9381076 | 0.00266469 | 0.002116182 |
| k__Bacteria;p__Proteobacteria;c__Gammaproteobacteria;o__Pasteurellales;f__Pasteurellaceae;g__Haemophilus;s__Haemophilus_pittmaniae | 0.00602611 | 0.938123949 | 0.002427922 | 0.000811809 |
| k__Bacteria;p__Actinobacteria;c__Actinobacteria;o__Actinomycetales;f__Micrococcaceae;g__Rothia;s__Rothia_mucilaginosa | 0.002438175 | 0.960618157 | 0.019478688 | 0.021382881 |
| k__Bacteria;p__Firmicutes;c__Clostridia;o__Clostridiales;f__Lachnospiraceae_XIV;Other;Other | 0.001655967 | 0.96754018 | 0.000199418 | 3.58E-05 |
| k__Bacteria;p__Proteobacteria;c__Betaproteobacteria;o__Neisseriales;f__Neisseriaceae;g__Neisseria;s__Neisseria_flavescens | 0.000447828 | 0.983116469 | 0.05927767 | 0.075591369 |
