# Supplementary material for: Characterization of the salivary microbiome in people with obesity
Source: PeerJ. 2018 Mar 16;6:e4458. doi: 10.7717/peerj.4458 (PMC5858547; doi:10.7717/peerj.4458)
Supplement: Table S8 — The relative abundances of 58 species were significantly different in the saliva samples of the obese group compared with that of the control group, in which 26 were over-represented in the obese group. (Kruskal–Wallis test, P < 0.05). [file peerj-06-4458-s010.docx]

|  | Species | normal_weight_mean | obesity_mean | *P*-value | FDR_P |
| --- | --- | --- | --- | --- | --- |
| Species over-represented in obesity group | s__Prevotella_nanceiensis | 0.00781037 | 0.017474279 | 0.006335522 | 0.039542395 |
|  | s__Fusobacterium_nucleatum_subsp._vincentii | 0.001856621 | 0.004039185 | 1.85E-05 | 0.000479429 |
|  | s__Peptostreptococcaceae_XIG-1_Eubacterium_sulci | 0.002164788 | 0.004065674 | 0.002718089 | 0.021390175 |
|  | s__Peptostreptococcus_stomatis | 0.001251882 | 0.002522018 | 0.002363689 | 0.020372748 |
|  | s__Solobacterium_moorei | 0.000755201 | 0.001506545 | 8.04E-05 | 0.001323102 |
|  | s__Mogibacterium_diversum | 0.000972043 | 0.001665186 | 0.000892384 | 0.009501265 |
|  | s__Alloprevotella_rava | 0.000133634 | 0.000745691 | 7.99E-05 | 0.001323102 |
|  | s__Catonella_morbi | 0.001021683 | 0.001564876 | 0.003741319 | 0.027087147 |
|  | s__Prevotella_scopos | 9.69E-06 | 0.000521852 | 0.005797415 | 0.038864155 |
|  | f__Leptotrichiaceae; Other | 3.15E-05 | 0.000359819 | 0.0026304 | 0.021390175 |
|  | s__Campylobacter_sp._oral_taxon_044 | 9.44E-05 | 0.000340902 | 0.006097543 | 0.03941626 |
| Species over-represented in normal weight control | s__Capnocytophaga_sp._oral_taxon_412 | 0.000461271 | 8.66E-05 | 1.72E-05 | 0.000479429 |
|  | s__Gemella_sanguinis | 0.000414615 | 0 | 6.04E-08 | 1.09E-05 |
|  | s__SR1_G-1_sp._oral_taxon_874 | 0.000422969 | 5.15E-06 | 1.98E-07 | 1.79E-05 |
|  | s__Leptotrichia_sp._oral_taxon_392 | 0.000887667 | 0.000169425 | 1.07E-05 | 0.000387844 |
|  | s__Cardiobacterium_hominis | 0.001026251 | 0.000287762 | 0.000431649 | 0.005208562 |
|  | g__SR1_G-1; Other | 0.000866561 | 8.27E-05 | 1.02E-05 | 0.000387844 |
|  | s__Bergeyella_sp._oral_taxon_322 | 0.002342527 | 0.001198847 | 0.002363689 | 0.020372748 |
|  | s__Oribacterium_asaccharolyticum | 0.001625281 | 0.000450563 | 0.006896287 | 0.041607596 |
|  | s__Leptotrichia_sp._oral_taxon_225 | 0.001527866 | 0.000267241 | 9.54E-05 | 0.001328755 |
|  | s__Staphylococcus_aureus | 0.001752189 | 4.22E-05 | 0.001696528 | 0.016161666 |
|  | s__Capnocytophaga_leadbetteri | 0.002549288 | 0.000727574 | 4.39E-06 | 0.000264693 |
|  | s__Corynebacterium_durum | 0.002621374 | 0.000727058 | 9.04E-05 | 0.001328755 |
|  | s__Gemella_morbillorum | 0.002846762 | 0.000795951 | 0.001615078 | 0.016161666 |
|  | g__Rothia; Other | 0.005055856 | 0.001407432 | 5.98E-05 | 0.001202046 |
|  | g__Fusobacterium; Other | 0.007118946 | 0.002966229 | 0.000295886 | 0.003825385 |
|  | s__Lautropia_mirabilis | 0.006915191 | 0.001910146 | 0.000505899 | 0.005722982 |
|  | s__Neisseria_mucosa | 0.014260833 | 0.003100194 | 0.00510344 | 0.035527795 |
|  | g__Streptococcus; Other | 0.029010229 | 0.016446529 | 0.003418059 | 0.025777862 |
|  | s__Haemophilus_parainfluenzae | 0.129984576 | 0.070706185 | 5.98E-05 | 0.001202046 |
