# Supplementary material for: Characterization of the salivary microbiome in people with obesity
Source: PeerJ. 2018 Mar 16;6:e4458. doi: 10.7717/peerj.4458 (PMC5858547; doi:10.7717/peerj.4458)
Supplement: Table S9 — Eighteen obesity-associated functional modules were obtained in the saliva samples using the FishTaco software. [file peerj-06-4458-s011.docx]

|  | Name | Class | Pathway |
| --- | --- | --- | --- |
| 0 : M00007 | Pentose phosphate pathway, non-oxidative phase, fructose 6P=> ribose 5P | Pathway module; Carbohydrate and lipid metabolism; Central carbohydrate metabolism | map01200  Carbon metabolism map01230  Biosynthesis of amino acids map00030  Pentose phosphate pathway |
| 1 : M00014 | Glucuronate pathway (uronate pathway) | Pathway module; Carbohydrate and lipid metabolism; Other carbohydrate metabolism | map00040  Pentose and glucuronate interconversions |
| 2 : M00020 | Serine biosynthesis, glycerate-3P => serine | Pathway module; Nucleotide and amino acid metabolism; Serine and threonine metabolism | map01200  Carbon metabolism map01230  Biosynthesis of amino acids map00260  Glycine, serine and threonine metabolism |
| 3 : M00026 | Histidine biosynthesis, PRPP => histidine | Pathway module; Nucleotide and amino acid metabolism; Histidine metabolism | map01230  Biosynthesis of amino acids map00340  Histidine metabolism |
| 4 : M00061 | D-Glucuronate degradation, D-glucuronate => pyruvate + D-glyceraldehyde 3P | Pathway module; Carbohydrate and lipid metabolism; Other carbohydrate metabolism | map00040  Pentose and glucuronate interconversions map00030  Pentose phosphate pathway |
| 5 : M00129 | Ascorbate biosynthesis, animals, glucose-1P => ascorbate | Pathway module; Nucleotide and amino acid metabolism; Cofactor and vitamin biosynthesis | map00040  Pentose and glucuronate interconversions map00053  Ascorbate and aldarate metabolism |
| 6 : M00179 | Ribosome, archaea | Structural complex; Genetic information processing; Ribosome | Structural complex; Genetic information processing; Ribosome |
| 7 : M00205 | N-Acetylglucosamine transport system | Structural complex; Environmental information processing; Saccharide, polyol, and lipid transport system | map02010  ABC transporters |
| 8 : M00219 | AI-2 transport system | Structural complex; Environmental information processing; Saccharide, polyol, and lipid transport system | map02010  ABC transporters |
| 9 : M00275 | PTS system, cellobiose-specific II component | Structural complex; Environmental information processing; Phosphotransferase system (PTS) | map00500  Starch and sucrose metabolism map02060  Phosphotransferase system (PTS) |
| 10 : M00277 | PTS system, N-acetylgalactosamine-specific II component | Structural complex; Environmental information processing; Phosphotransferase system (PTS) | map00052  Galactose metabolism map02060  Phosphotransferase system (PTS) |
| 11 : M00283 | PTS system, ascorbate-specific II component | Structural complex; Environmental information processing; Phosphotransferase system (PTS) | map00053  Ascorbate and aldarate metabolism map02060  Phosphotransferase system (PTS) |
| 12 : M00308 | Semi-phosphorylative Entner-Doudoroff pathway, gluconate => glycerate-3P | Pathway module; Carbohydrate and lipid metabolism; Central carbohydrate metabolism | map01200  Carbon metabolism map00030  Pentose phosphate pathway |
| 13 : M00318 | Iron/zinc/manganese/copper transport system | Structural complex; Environmental information processing; Metallic cation, iron-siderophore and vitamin B12 transport system | map02010  ABC transporters |
| 14 : M00361 | Nucleotide sugar biosynthesis, eukaryotes | Functional set; Metabolism; Nucleotide sugar | map00520  Amino sugar and nucleotide sugar metabolism |
| 15 : M00434 | PhoR-PhoB (phosphate starvation response) two-component regulatory system | Functional set; Environmental information processing; Two-component regulatory system | map02020  Two-component system |
| 16 : M00448 | CssS-CssR (secretion stress response) two-component regulatory system | Functional set; Environmental information processing; Two-component regulatory system | map02020  Two-component system |
| 17 : M00550 | Ascorbate degradation, ascorbate => D-xylulose-5P | Pathway module; Nucleotide and amino acid metabolism; Cofactor and vitamin biosynthesis | map00053  Ascorbate and aldarate metabolism |
