# Supplementary material for: Characterization of the salivary microbiome in people with obesity
Source: PeerJ. 2018 Mar 16;6:e4458. doi: 10.7717/peerj.4458 (PMC5858547; doi:10.7717/peerj.4458)
Supplement: Supplemental Information 2 — An empty copy of the questionnaire we used. [file peerj-06-4458-s014.doc]

**Questionnaire**

Dear sir/madam：

Microbes are extremely small sized organisms, which inhabit almost every corner of our planet, including human’s oral cavity. Oral microbiome vary from one individual to another. This variation is said to play a role in certain kinds of diseases. Our team has been working on this topic and this survey is designed for the very purpose. Data collected would then be processed via certain statistic strategies, and we promise no divulgence to any individual or organization. Our study is essentially based on your answers, thus we will highly appreciate your honesty as well as any other offered support.

Please answer each question according to your own situation. Mark the choice that fits you most with “√”, and then write the number before the chosen answer in the □ on the right. Again, we give much of our appreciation to your kind support.

| 1. **General information**   1.01、Your gender ： Male=1 Female=2  1. 02、Your date of birth：Year____ Month ____  1. 03、Your nationality：Han=1 Other=2（Please specify）______ | □    □ ______ |
| --- | --- |
| 1.04、Height：____ cm Weight：____ kg  1.05、Profession： Student=1 Teacher=2 Medical worker=3  Public official=4 Enterprise Employee=5  Self-employed=6 Migrant worker=7  Retiree=8 Unemployed=9  Other=10（Please specify）________  1.06、Your educational level： Primary school or lower=1  Junior high school=2  Senior high or technical secondary school=3  Undergraduate degree or junior college=4  Graduate degree or higher=5 | ，  □  □ |
| 1.07、Have you received any anti-obesity treatment recently ?(E.g. diet pills, weight-loss surgery, etc.)  No=0 Yes=1（Please specify）  1.08、Have you ever been a regular smoker？：  No=0 Yes=1 (Please continue):  (1) For how many years have you been smoking? ____year(s)  How many cigarettes a day on average?____  If you are an occasional smoker. How many boxes of cigarettes per year? ____box(es)  If quitted. How many years it has been since you stopped smoking? ____year(s)  How many cigarettes a day before quitting? ____.  How many years had you smoked? ____year(s).  1.09、Have you ever been a drinker?  No=0 Yes=1 (Please continue)  You’re a frequent user of:  Liquor=0 (Alcoholicity higher than 38%)  Non-liquor=1 (Alcoholicity lower than 38%)  Average amount of daily consumption. ____tael/per day. | □  □  ，    ，  □  □ |
| 1. **Physical status**   2.01、Have you suffered from any syndromes or diseases listed below? (Please mark every fitting one with √ ).  **Diabetes mellitus** No=0 Yes=1 Unknown=2  **Hypertension**  No=0 Yes=1 Unknown=2  **Hyperlipidemia** No=0 Yes=1 Unknown=2  **Gastrointestinal diseases** (E.g. CG, GU, etc.)  No=0 Yes=1  **Cardiovascular and cerebrovascular diseases**  No=0 Yes=1  **Other systemic diseases** (Please specify) ________  2.02、Are you suffering from any infectious diseases listed below? (Please mark every fitting one with √ ).  Hepatitis or carrying the virus=1  Pulmonary tuberculosis=2  Bacillary dysentery=3  AIDS=4  Others=5 (Please specify) ______  None=0  2.03、Have you ever been a long-term consumer of certain medicines?  No=0 Yes=1 (Please specify) ______  2.04、During the last three months, have you ever taken any medicine listed below? (Please mark every fitting one with √ )  Antibiotics=1 (E.g. cephalosporin, metronidazole, etc.)  Hormone drugs=2  Immunosuppressive drugs=3  Others=4 (Please specify) ______  None=0  2.05、(This one is for female) Are you pregnant?  No=0 Yes=1  2.06、(This one is for female) Is it your menstrual period?  No=0 Yes=1 | □  □  □  □  □  □  □，  □  □  □ |
| 1. **Oral health maintenance**   3.01、How often do you brush your teeth?  ≥2 times/day=1 1 time/day=2 2-3 times/week=3 Never=4  3.02、How often do you use floss or interdental brush?  ≥1 time/2 days =1 1-2 times/week=2 Occasionally=3 Never=4  3.03、How often do you use mouthwash?  ≥1 time/2 days =1 1-2 times/week=2 Occasionally=3 Never=4  3.04、During the last three months, have you ever received any dental treatment as listed below?  Clearing=1 Fillings=2 Denture/partial denture=3  Braces, orthodontic treatment, or bite adjusted =4 None=0 | □  □  □  □ |

Your name： Your phone number：

Your email address：

We sincerely appreciate your time as well as your support. Best regards to you and your beloved ones.
